# Synergy of climate change with country success and city quality of life

**DOI:** 10.1038/s41598-023-35133-4

**Published:** 2023-05-15

**Authors:** Arturas Kaklauskas, Ajith Abraham, Loreta Kaklauskiene, Ieva Ubarte, Dilanthi Amaratunga, Irene Lill, Virginijus Milevicius, Ulijona Kaklauskaite

**Affiliations:** 1 Vilnius Gediminas Technical University, Vilnius, Lithuania; 2 FLAME University, Pune, India; 3 University of Huddersfield, Huddersfield, UK; 4 Tallinn University of Technology, Tallinn, Estonia; 5 Vilnius University, Vilnius, Lithuania

**Keywords:** Climate sciences, Ecology, Environmental sciences, Natural hazards, Planetary science, Health care, Energy science and technology, Mathematics and computing, Climate-change adaptation, Climate-change impacts, Climate-change mitigation, Climate-change policy, Energy and society, Environmental economics, Environmental impact, Psychology and behaviour, Socioeconomic scenarios, Sustainability

## Abstract

Most people around the world have felt the effects of climate change on their quality of life. This study sought to achieve the maximum efficiency for climate change actions with the minimum negative impact on the well-being of countries and cities. The Climate Change and Country Success (C^3^S) and Climate Change and Cities’ Quality of Life (C^3^QL) models and maps of the world created as part of this research showed that as economic, social, political, cultural, and environmental metrics of countries and cities improve, so do their climate change indicators. For the 14 climate change indicators, the C^3^S and C^3^QL models indicated 68.8% average dispersion dimensions in the case of countries and 52.8% in the case of cities. Our research showed that increases in the success of 169 countries saw improvements in 9 climate change indicators out of the 12 considered. Improvements in country success indicators were accompanied by a 71% improvement in climate change metrics.

## Introduction

Millions or billions of people are vulnerable to the potential harm brought by climate change to their lives and livelihoods^1^. Different countries are, however, using different climate change policies, programs, and measures, and their achievements also differ. Cities play a central role in determining the sustainability of human development^2^. The Paris Agreement on Climate Change and the UN’s Sustainable Development Goals (SDGs) call for all countries to seek deep transformations through concerted efforts by civil society, governments, business, and science^3^. These decisions, however, should be mathematically sound and rational. Fuso Nerini et al.^4^ reviewed the available evidence, and their structured review shows that 16 SDGs may suffer negative impacts due to climate change, while efforts to mitigate climate change can strengthen all 17 SDGs but also have a negative impact on endeavors to achieve 12 of them. Synergies between some targets should be promoted and trade-offs between other targets mediated to achieve the SDGs^5^.

Climate impacts as a factor of environmental pillar can in some cases be measured in terms of the indicators of social and economic pillars of sustainable development. Many different effects of climate are difficult to quantify or have not been sufficiently examined and are therefore not included in current evaluations of the risks posed by climate change for people’s lives and livelihoods^1^. Many different quantitative models have been developed to inform policy decisions, but increases in model complexity and coverage often do not make the model more relevant or accurate^6^. In the past, climate researchers often employed scenarios that combined broad narratives and quantitative model-based projections^7^. Many projects have also been based on a narrative approach^8^. In attempts to address human behavior in climate models, social climate models (SCMs) have emerged as a new class of model^9^. Hornsey and Lewandowsky^10^ analyzed how policy-making in areas such as the assignment of responsibility for supply chain emissions, carbon taxing, and natural-disaster insurance markets can use insights from behavioral science. Many other quantitative, qualitative, and integrated models to handle various climate change areas have been developed around the world^11, 12^, and several researchers have taken integrated sustainable development^13, 14^ approaches to analyze climate change^15^ and looked at multiple dimensions^16^. The achievement of all 17 SDGs at once will require the ability to make plans and decisions that determine and utilize synergies, which are positive interactions, and limit trade-offs, which are negative interactions, between the goals and their 169 targets^17^.

Changes in energy systems are necessary, and Fuso Nerini et al.^18^ identified 113 targets for which action is required; they also published evidence that 143 targets (65 trade-offs, 143 synergies) and endeavors to achieve SDG 7 are linked. Vinuesa et al.^19^ used a consensus-based expert elicitation process and determined that artificial intelligence (AI) could help achieve 134 targets across all of the SDGs, but may have a negative impact on 59 targets.

Most of the SDGs are in some way related to cities. Goal 6 calls for clean water and sanitation and goal 7 for clean and affordable energy. Goal 11 focuses on sustainable communities and cities; goal 8 on economic growth and decent work; goal 9 on infrastructure, innovation and industry; goal 12 on responsible production and consumption; and goal 13 on climate action. All of these aspects are important to sustainable cities^20^. The framework proposed by Fuldauer et al.^21^ conceptualized the complex influences flowing in both directions between climatic impact drivers, all 169 SDG targets, and a holistic set of 22 socio-economic sectors and ecosystems. They applied the framework globally and showed that near-term sectoral risk related to climate change is a threat to all 169 SDG targets. Multiple criteria decision-making (MCDM) can help model the multifunctionality, reliability, and consequences of potential maladaptation of nature-based solutions and engineering-based adaptation options, with the SDG targets as one of the contexts^21^.

Decisions about which risks should not be tolerated and which values of which people should be prioritized will affect the extent of climate change and the response of societies^22^. Each action related to adaptation will come from a decision made with values in mind: what we should preserve, what we can allow to change unguided, and where purposeful changes need to be made^23^. Communities should give serious thought to the option of retreat as a transformative climate adaptation^22^. Statistical and MCDM analyzes were used to assess the impact on climate change indicators of the success of countries, 169 in total, and the quality of life in cities, 238 in total. Three key hypotheses were proposed and validated for this study:

- *Hypothesis 1*. Improvements (synergistic effects) in climate change indicators and climate change mitigation, adaptation, and resilience actions can be achieved for the countries and cities considered without being limited to environmental, economic, and other traditional means. Other less explored areas, such as reducing corruption, improving human development, ensuring gender equality, improving democracy, and happiness can also contribute.
- *Hypothesis 2*. A country’s success, its indicator system, and the climate change indicators are interrelated. A city’s quality of life and climate change indicators are also interconnected. With a different set of indicators of a country’s success, a city’s quality of life, and climate change, the boundaries of 169 countries and 238 cities in the seven clusters remain similar. The second hypothesis is based on the integrated sustainability perspective, which considers a significant spillover effect between the quality of life as a factor of the social development pillar and the climate change as an environmental development pillar.
- *Hypothesis 3*. The increasing success of a country and improvement in its indicators are usually accompanied by an improvement in climate change indicators. Improving quality of life in cities also generally goes together with improvement in climate change indicators. In other words, the third hypothesis more specifically analyzes the synergistic character of the spillover effects between the quality of life and climate actions improvement from the integrated sustainability perspective.

All three hypotheses are based on the analysis results related to the Climate Change and Cities’ Quality of Life (C^3^QL) and Climate Change and Country Success (C^3^S) world maps and models, which were used to validate the three hypotheses proposed in this study. Many studies present lists of factors important for climate change mitigation, adaptation, and resilience, as well as their statistical dependences. This research applies statistical, MCDM, and recommender methods to show numerically how a country’s success and a city’s quality of life affect climate change indicators.

The main aim of this study is to achieve the most synergistic effect: the maximum efficiency for climate change actions with the minimum negative impact on the country’s success and the quality of life in its cities. Our integrated approach to the way the country-level social, economic, environmental, and political context affects climate change indicators is one key aspect that makes this research different from other existing studies^11–14^ that have looked at only a few of these indicators at a time. Likewise, the analysis of seven social, four economic, one environmental, and three political indicators across 169 countries has indicated synergies with climate change indicators at 68% (57 out of 84), 73% (35 out of 48), 75% (9 out of 12), and 75% (27 out of 36) respectively (see Supplementary Table S4b). Our study is different from similar research due to three key innovative elements. The first is that the models and maps that were created by the RECASTM (*R*ecommendation m*e*thod for *c*limate *c*hange mitigation, *a*daptation and resilience *st*atistical and *m*ulticriteria analysis) method on the basis of multicriteria and statistical analysis across 169 and 173 countries and 238 cities show a link between climate change, cities’ quality of life and a country’s success. Second, as part of this study, Climate Change and Country Success (C^3^S) and Climate Change and Country Success (C^3^QL) models and maps have been developed, and they demonstrate that as the progress of countries’ political, cultural, and social indicators also have a synergistic effect on their climate indicators. Third, we found that environmental, social, cultural, political, and economic sustainability indicators move collectively in a similar direction. These three innovative elements of the STRICT method are demonstrated within the article.

## Methods

The Climate Change, Country Success and Cities’ Quality of Life (C^3^S-QL) sustainability framework has been developed using the EEA Conceptual framework for urban environmental sustainability^24, 25^ as its basis. We describe C^3^S-QL sustainability framework as having three pillars (or dimensions)^13, 14^. The framework includes six stages. In the first stage, stakeholders can make analysis using various lenses (smart, green, inclusive, circular, resilient, healthy or low-carbon city) individually or their combinations. Country perspectives are determined likewise.

In the second stage, multiple criteria and statistical analysis was used to analyze the enabling factors in countries and cities, which are high-level forces such as:

- culture (two dimensions of the 2020 Inglehart–Welzel Cultural Map of the World^26^);
- finance (GDP per capita, GDP per capita in PPP, ease of doing business ranking, and economic decline index);
- governance (freedom and control, economic freedom, and democracy index); and
- social (corruption perceptions index, human development index, global gender gap, happiness index, unemployment rate, healthy life expectancy, and fragile state index).

In the third stage, multiple criteria and statistical analysis was used to investigate the context of countries (Supplementary Table S1) and cities (Supplementary Table S2), meaning the set of indicators, their values and weights).

In the fourth stage, the synergies and trade-offs related to climate change indicators were analyzed, as well as sustainability spillover effects related to the economic, environmental, and social aspects. In the fifth stage, synergies and trade-off show the ability of swapping the measurements between different sustainable development pillars. The fifth stage aims to estimate the elasticities of sustainability and success for 169 countries to show the synergetic and trade-off character of country success and climate change. In this stage, the effects of the social, environmental, and economic sustainable development pillars on climate change are estimated and compared, as are their potential spillover effects (Supplementary Table S4b).

In the sixth stage, we have identified city building blocks (housing quality^27^, public open space^28^, built environment quality^29^, energy efficiency^27^, renewable energy^30^), which, depending on the context and enabling factors, will be the inputs essential to the transition to city quality of life and country success.

The C^3^S-QL sustainability framework was used as a basis to develop the *R*ecommendation m*e*thod for *c*limate *c*hange mitigation, *a*daptation and resilience *st*atistical and *m*ulticriteria analysis (the RECASTM method).

This method seeks to mitigate climate change with maximum efficiency and minimum negative impact on the needs of countries and cities. Also, this study pursued to determine the country and city indicators that would, as they are improved, ensure maximum efficient handling of climate change mitigation, adaptation, and resilience issues.

Scores of different national climate policy alternatives can then be generated and analyzed against many different criteria and the most effective options selected. The result of this research is the RECASTM method, which is based on our European Patent application (EP 4 020 134 A1). The primary goal of the RECASTM method is to maximize country success and city quality of life and minimize the impacts of climate change. The research includes the following steps: (1) development and testing of three hypotheses; (2) data collection and conducting a multi-criteria study of 169 countries and 238 cities by applying the INVAR method; (3) correlation analysis of 238 cities for 9 quality of life indicators, 169 countries for 32 success (17 control cluster metrics and 15 experimental cluster metrics) indicators, and their 14 (12 countries and two cities) quantitative and qualitative climate change indicators; (4) development of C^3^QL and C^3^S models; (5) creation of the C^3^QL and C^3^S maps to visualize how the success of 169 countries and the quality of life of 238 cities affected improvements in their climate change indicators; (6) calculating the elasticity coefficient for each independent variable; (7) analysis of climate change synergies and trade-offs; and (8) development of national and city climate change policy options, using MCDM and regression analysis to identify rational solutions.

This study’s three key hypotheses were proposed and validated during the first step. To comprehensively assess the quality of life in 238 cities and the success of 169 countries and to link this to climate change, we developed a comprehensive criteria framework in the second step. The city criteria framework included nine quality of life and two climate change criteria. The country criteria framework consisted of 32 country success and 12 climate change criteria, as well as two Inglehart–Welzel 2020 Cultural Map of the World^26^ dimensions. The 169 nations 32 country success indicators analyzed were allocated to a control cluster and an experimental cluster. These two clusters consist of integrated sustainability set of economic, social, political, and environmental metrics. The control cluster dataset covers 17 metrics (GDP per capita, GDP per capita in PPP, ease of doing business ranking, corruption perceptions index, human development index, global gender gap, happiness index, environmental performance index, freedom and control, economic freedom, democracy index, unemployment rate, healthy life expectancy, fragile state index, economic decline index, and two calculated results of all this metrics—country success and country success priority of 169 countries). The experimental cluster dataset covers 15 metrics (government effectiveness, civil liberties, the global sustainable competitiveness index, human rights and rule of law index, wealth per adult, ecological footprint per capita, environmental health, air quality, PM_2.5_ exposure, life expectancy at birth, death rates from air pollution, positive peace index, and three calculated results—country success, country success priority, and country national competitiveness degree of 173 countries). We used the same indicators (GDP per capita, GDP per capita in PPP, ease of doing business ranking, corruption perceptions index, human development index, global gender gap, economic freedom, democracy index, unemployment rate, fragile state index, and economic decline index) dataset for all countries of the control and experimental clusters. These indicators, along with some other metrics (government effectiveness, civil liberties, the global sustainable competitiveness index, human rights and the rule of law index, wealth per adult, and population growth indicators), were used to calculate the country's success (173 countries, 17 indicators) and are displayed in the control cluster and not repeated in the experimental cluster. Supplementary Table S1 displays the full list of representative indicators, indicators’ baseline data, source links, and results of the MCDM analysis of the indicators for the success of 169 countries and climate change. The quality of life and climate change indicators for 238 cities are presented in Supplementary Table S2.

There has been recent substantial development and application of MCDM methods. According to the various techniques studied (ELECTRE, the multi-attribute utility theory, PROMETHEE, data envelopment analysis, AHP, goal programming, TOPSIS, a combination of techniques), the perceived benefits of the INVAR technique are that: it has a comprehensible rationality; it is easy to apply in steps 1–5 and results are obtained relatively quickly; it is very practical for use in steps 5–10; it analyzes both quantitative and qualitative data and integrates weights; and it evaluates numerous dimensions. The main drawback of the INVAR technique is that, compared to other methods, the calculation cycles in steps 6, 7, and 10 are lengthier and more complicated. The main innovations of the INVAR technique have been presented in MCDM studies in a few countries^31, 32^ and cities^28^. The quality of life and success of the 238 cities and 169 countries analyzed applying this INVAR technique (Supplementary Sect. 3) depends directly and proportionately on a system of criteria that characterizes them adequately, and on the weights and values of the indicators^33^ (Supplementary Tables S1 and S2). Experts create the system of criteria and calculate criteria values and initial weights; politicians and other stakeholders can then make adjustments to this information so that it fits their purposes. When any available alternatives are assessed, the results will fully reflect the initial data provided jointly by experts and stakeholder groups.

After examination of the cities and countries being compared, the outcomes are obtainable in a matrix, the columns showing *n* cities/countries, and the rows showing the comprehensive system of criteria that characterize these cities/countries. The system of criteria includes criteria names, their measurement units, values and weights, and the labels that mark each criterion either as minimizing (a lower value is denoted by target achievement, as in the case of the unemployment rate, the economic decline index, the fragile state index) or maximizing (a higher value is denoted by target achievement, as in the case of economic freedom, the democracy index).

Before rational decisions on climate change resilience, mitigation, and adaptation can be made, MCDM assessment of their potential alternatives is required. Quantitative, qualitative and integrated approaches are used for this purpose. We used statistical climate change indicators (energy use rank, ND-GAIN index, EPI climate change, EPI greenhouse gas emissions per capita, EF carbon footprint, CO_2_ emissions from fossil fuel combustion and cement production, CO_2_ emissions embodied in imports, carbon pricing, global gridded model of carbon footprints, and BASIC GHG emissions data for C40 cities) and subjective-survey indicators (climate perceptions, public belief in the climate emergency, respondents by country who say we should do everything necessary) in our research. The research determined a moderate median correlation (r = 0.491) between statistical and survey-based climate change indicators. Similar research findings have been presented by Jones and D’Errico^34^, who determined a moderate correlation between data related to objective and subjective approaches.

We performed a correlation analysis of 238 cities for 9 quality of life indicators, of 169 countries for 32 success (17 control cluster and 15 experimental cluster metrics) indicators, and their 14 (12 countries and 2 cities) quantitative and qualitative climate change indicators in the third step (Supplementary Table S3).

The fourth step is to build 12 C^3^S regression models and calculate their effect size indicators (Supplementary Tables S4–S8). IBM SPSS V.26 software was used to develop the C^3^S and C^3^QL models.

**Figure 1:**
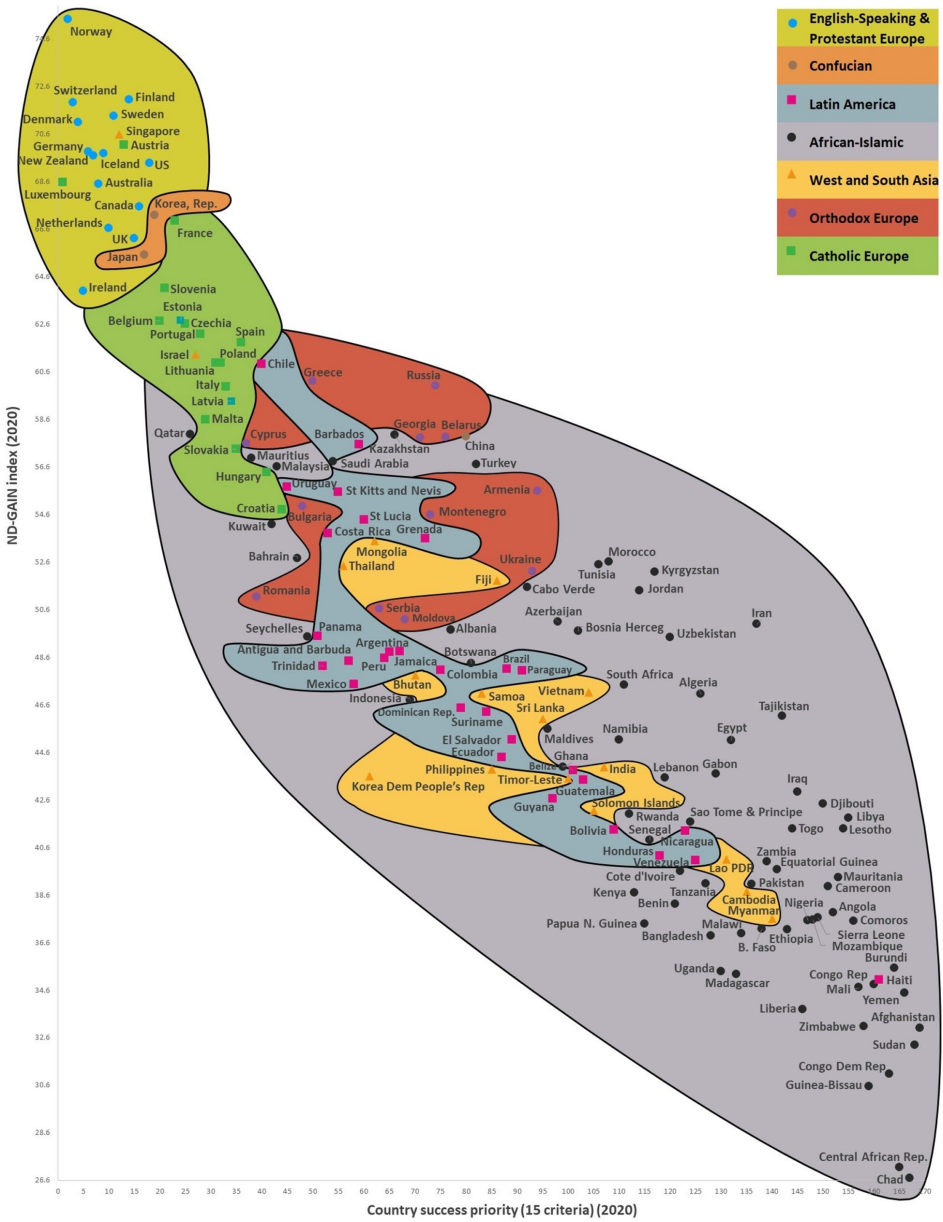
The C^3^S ND-GAIN World Map. The C^3^S ND-GAIN world map shows 163 countries grouped in clusters based on the values found along the y-axis (ND-GAIN index) and the x-axis (country success priority). Each country mapped along the x-axis has its priority influenced by the system of indicators, context and by their values and significance. As a country’s success improves, it moves closer to the left side of the map. Improvements in the ND-GAIN country index—which reflects a country’s vulnerability to climate change and other global issues, as well as the country’s readiness to improve resilience—push countries up in the map. The location of countries on the map, then, can be used to forecast how they respond to the evolving situation of their vulnerability to climate change. The way countries move from the bottom right to the top left section of the map shows their transition towards the country’s vulnerability to climate change and other global challenges in combination with its readiness to improve resilience. ND-GAIN country index aims to help governments, businesses, and communities prioritize their investments better to respond more efficiently to the immediate global challenges waiting ahead^78^. A successful country evidently has a greater chance of becoming less vulnerable to climate change. The C^3^S ND-GAIN world map is a systematic visualization of tight groups of nations that are interconnected and polarized. The clusters are independent of both the quantity of their descriptive variables and the countries selected for nexus analysis. Climate change indicators on the y-axis and national success and city quality of life indicators on the x-axis are two predominant dimensions and reveal the differences between different clusters and societies. The city quality of life and country success indicators and context describe the shared values characteristic to a nation cluster. Eight clusters used in the Inglehart–Welzel 2020 World Cultural Map^26^, namely Catholic Europe, English-speaking & Protestant Europe, Orthodox Europe, Confucian, West & South Asia, African-Islamic, and Latin America, are also used in the C^3^S maps. We generated the map using our RECASTM method and the Windows 10–11 Office 2016–2021 Excel software.

**Figure 2:**
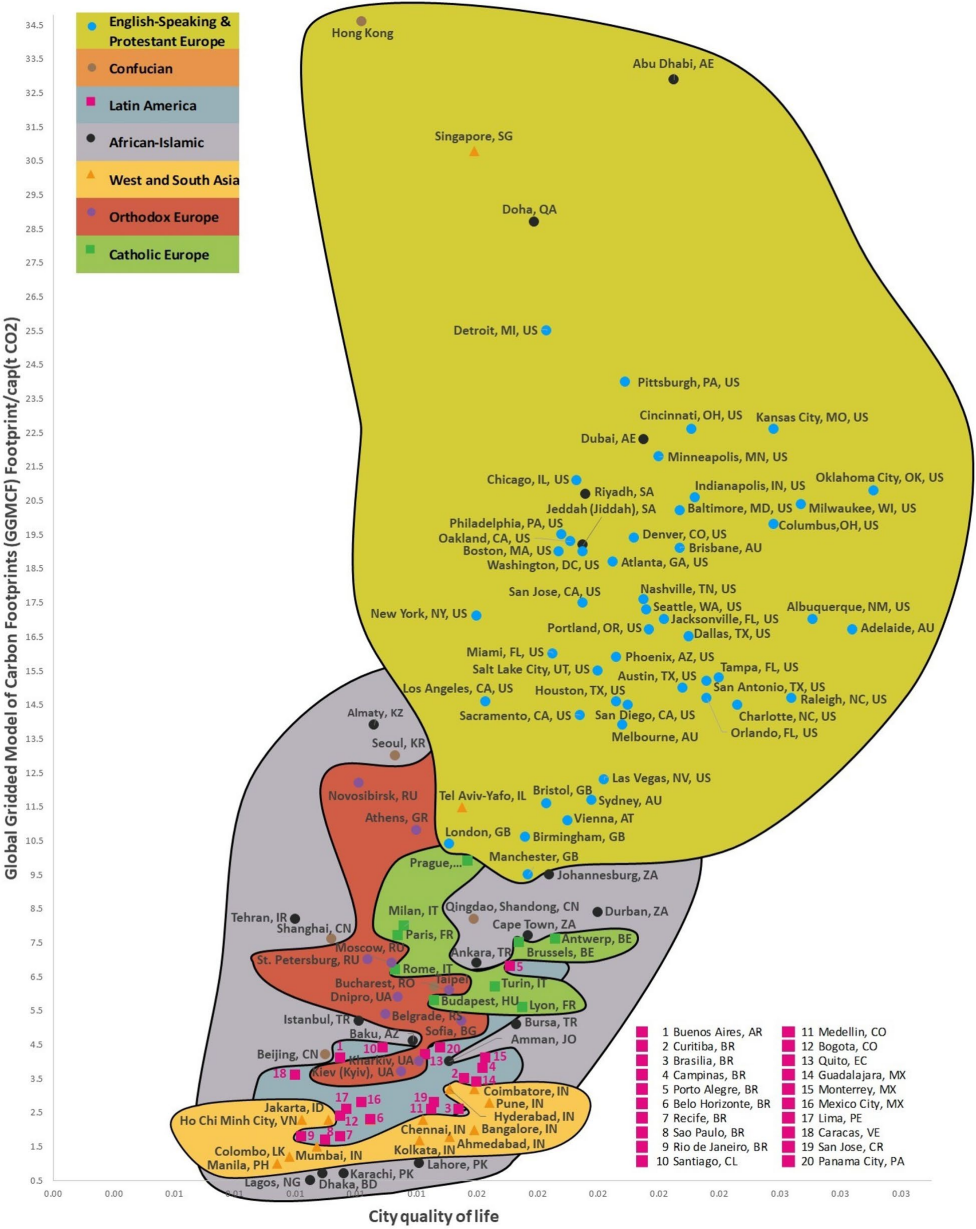
World Map of City Quality of Life and Global Gridded Model of Carbon Footprints. The Global Gridded Model of Carbon Footprints (GGMCF) offers per capita estimates of carbon footprints across 189 countries in a globally consistent and spatially resolved form and incorporates currently available subnational models for China, Japan, the EU, the UK and the US. On the map, the x-axis dimension shows the quality of life of individual cities and the y-axis dimension displays the carbon footprint per capita of each city. Nine variables were used to calculate the dimensions of city quality of life shown on the map. This map includes eight country clusters related to the classifications used in the 2021 World Values Survey and the 2020 World Cultural Map^26^. The map in this figure shows one dimension of the full spectrum from the integrated sustainability approach taken in this research; this dimension illustrates that city quality of life and climate change indicators make a single whole that combines a variety of synergies and trade-offs. The way countries move from the bottom left to the top right section of the map shows their transition towards the sustainability of urban carbon footprints. GGMCF shows that meaningful impact on national and global emissions is possible through local action at national and city level^45^. As illustrated by this map, for instance, we see that cities located in the most successful countries use the most advanced risk reduction and prevention, preparation, response, and recovery measures, but they also use considerable amounts of resources to ensure their high quality of life and, therefore, their position on the map is not very good in terms of curbing climate change. Note that the average correlation (r = 0.669) of this map’s x and y dimensions confirms the second hypothesis. We generated the map using our RECASTM method and the Windows 10–11 Office 2016–2021 Excel software.

**Figure 3:**
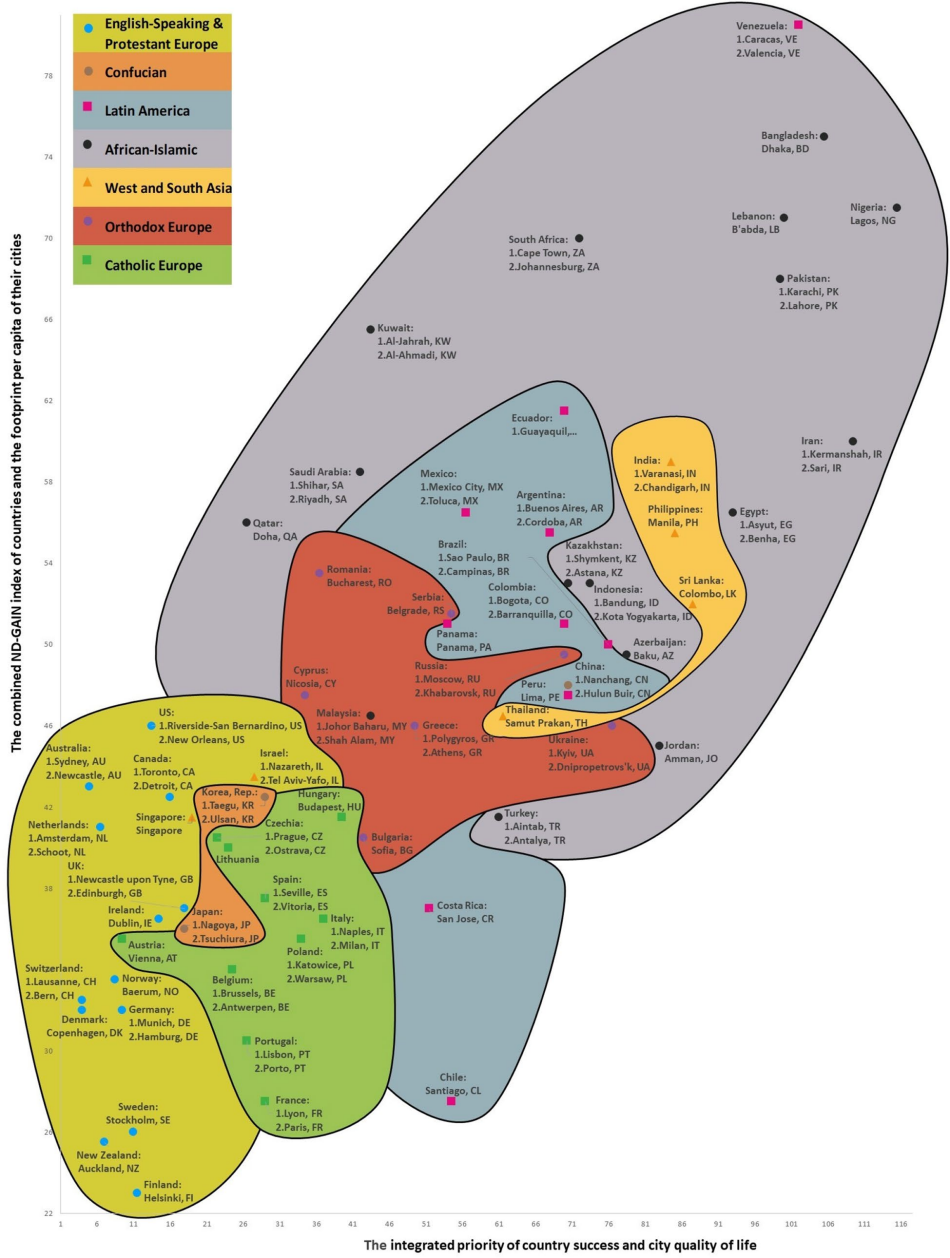
Integrated World Map of Climate Change, Country Success, and City Quality of Life. This snippet illustrates the relationship between the integrated success of 65 countries and the quality of life of their cities, 210 in total, plotted on the x-axis dimension, and the combined ND-GAIN index of countries and the carbon footprint per capita of their cities, 713^45^ in total, plotted on the y-axis dimension. Fifteen available success indicators from 169 countries were used to calculate the success of each country. The map also shows which city in each country has the lower value, which indicates target achievement (1), and a higher value, which indicates target achievement (2) carbon footprint per capita (tCO_2_/cap). The map shows 102 cities out of a total of 713 analyzed in this research. The way countries move from the top right to the bottom left section of the map shows their transition towards integrated climate change, and city quality of life sustainability. The map shows that a country’s success and its cities’ quality of life and the climate change indicators are interrelated (r = 0.777) and that (1) the increasing success of a country and the improvement of city quality of life are usually accompanied by (2) improvement in climate change indicators and context. This, then, has again validated the second and third hypotheses. We generated the map using our RECASTM method and the Windows 10–11 Office 2016–2021 Excel software.

We created the C^3^QL, C^3^S, and integrated maps to visualize how the success of the 169 countries and the quality of life of the 238 cities affected the improvement of its climate change indicators in the fifth step (Figs. 1, 2, 3). Steps 4 and 5 involved the development of the C^3^QL, C^3^S, and the related integrated models and maps, which validated an integrated sustainability approach to climate change (“Results”).

In the sixth step, we calculated the elasticity coefficient for each independent country's success variables to measure the effect of changes in each independent variable included in the initial model on the dependent climate change variable. The coefficient indicates by what magnitude the value of the dependent variable changes when the value of an independent variable goes up by 1%. Table 2 lists the results of these calculations.

We analyzed climate change indicators synergies and trade-offs in the seventh step. The synergies and trade-off results matrices are displayed in Supplementary Table S4b. The colors represent the synergies (green) and trade-offs (pink) observed in the climate change indicators when a nation’s success increases by 1%. Understanding both negative (trade-offs) and positive (synergies) interactions is very important in pursuing integrated research and decision-making toward action on climate change. In Kroll et al.’s^35^ opinions, these interactions can be either trade-offs, in which progress in one goal negatively impacts progress in another, or synergies, in which progress in one goal helps with progress in another.

In the eight step, a two-step process of mapping and MCDM and regression analysis of national and city climate change policy options was carried out to identify rational solutions. All countries have very specific micro, meso, and macro environments that largely determine which measures (clean energy, energy conservation, carbon taxation, natural-disaster insurance markets, etc.) will have the greatest impact on improving climate change performance. In the opinion of Mach and Siders^36^, societies, depending on evolving values they see as priorities (e.g., environmental health, public safety, economic growth, cultural heritage, or social justice), will transform in certain ways and choose certain tools. The results obtained in the seventh step therefore differ for each country. For example, the seventh step calculations are presented below for a sample of countries.

Traditionally, for businesses and communities, a crucial input into policies and long-term planning processes is the economic evaluation of the risks posed by climate change. Integrated cost–benefit assessment models offer a highly aggregated approach and have therefore traditionally been used as a means to identify climate policies that can maximize global welfare through intertemporal optimization. Optimization is not, however, required in economic assessments of scenarios^1^. Resolution while studying optimal mitigation can also be increased by making improvements in stochastic optimization techniques^37^. We used the RECASTM method in this study.

The following supplementary study purposes were selected: (1) to outline the influence of a country’s success and a city’s quality of life indicators on climate change factors; and (2) to propose interested parties’ recommendations concerning strategies for improving climate change metrics. In light of the study outcomes, we discuss several strategies for improving climate change values, particularly for low indicators.

To identify the key social, economic, environmental, and political indicators that make the most significant impact on all 12 selected quantitative and qualitative climate change indicators for 169 countries, we assumed that the most significant impacts would be seen among the countries' success indicators that show the most considerable cumulative effect (Q_j_, P_j_, N_j_) on the improvement of climate change mitigation, adaptation and resilience actions.

Supplementary Table S9 shows the decision-making matrix and RECASTM method that help determine the most efficient ways to make customized improvements to countries’ climate change indicators. Twelve models for C^3^S with 15 independent variables are analyzed for this purpose. The decision-making matrix therefore consists of 12 similar parts, one for each specific model (C^3^S) to be analyzed. The first part concerns the first C^3^S (energy use rank) model, the final one the twelfth (carbon pricing score at EUR60/tCO_2_). All alternatives are assessed against a system of nine criteria. Rational ways to improve the climate change mitigation, adaptation, and resilience actions are determined by analyzing 15 independent variables to establish which of these 15 social, economic, environmental, and political indicators of the control cluster have the most significant cumulative effect (Q_j_, P_j_, N_j_) on the improvement of climate adaptation and mitigation actions.

Investigation of the best worldwide practices and the available scientific knowledge shows that C^3^S models need the processes of effect size (q_j_) and weight of independent variables (Q_j_). A variety of objective, subjective, and integrated weighting methods can be used to that end. The steps below can help users optimize their countries’ climate change indicators:

- Assign certain weights to their needs for C^3^S (regression models), and
- Set weights for each success factor individually

To determine weights (q_j_ and Q_j_), experts should consider the country’s context, practical benefits, and indicators with low values. Many researchers^38^ support the statement that it is usually easier or less costly to improve, by the same degree, an indicator with low value than an indicator with high value. Having established the cumulative effect (Qj, Pj, Nj) of 15 country success indicators in terms of improvement of climate change mitigation, adaptation, and resilience actions, we recommend performing a thorough analysis of these top country success indicators. The identification of country-specific success factors that should be prioritized for improvement allows for more detailed regression^39^ and MCDM^40^ analysis of these factors.

Although the RECASTM method (Supplementary Table S9) analyzes nine statistical indicators that can help determine country success factors with the most significant impact on improving climate indicators, our case study looked at three integrated regression metrics (Supplementary Table S4):

- A factor’s degree of impact (If), which is a function of the correlation coefficient (r), correlation weight (p_c_), the coefficient β of the dependent variable, and the weight of the t-statistics of coefficient B (p_t_) in the regression model, If = f(r, p_c_, β, p_t_); the weights of the impacts in the regression models were calculated for each dependent variable and the results are listed in Supplementary Table S4a;
- A percentage increase or decrease of a climate change indicator caused by a 1% increase in a country’s success (P_id_, Supplementary Table S4b); and
- The independent variables of country success that explain a specific share of dependent climate change variable under analysis (R^2^, Supplementary Table S4c). The RECASTM method is applied in our case study and designed to help its users find the most efficient ways to analyze specific countries and improve their climate change indicators in a customized way.

For this purpose, 12 climate change indicators with the three regression metrics (If, P_id_, R^2^) mentioned above are analyzed. The decision-making matrix thus consists of three similar parts, one for the analysis of each specific regression metric to see which of the country success indicators have the greatest cumulative effect (Q_j_, P_j_, N_j_) on improving climate change mitigation, adaptation, and resilience actions (Supplementary Table S4).

Upon identifying the most efficient country success indicators (Supplementary Table S4), their further detailed analysis with the INVAR method is proposed to determine specific micro-, meso-, and macro-level variables at the country level that drive the biggest improvements in the 12 climate change indicators in 169 countries.

The proposed RECASTM method can be a tool for policymakers, legislators, businesses, and communities, with significant consequences and implications. By applying these C^3^S, C^3^QL, and integrated models for each country and city and building on existing global best practices^41–43^ country- and city-specific recommendations can be made. The results are used to propose recommendations to stakeholders concerning the best policy plans for improving climate change values, especially for indicators with low values that are best suited to the target countries. Phillis et al.^38^ have suggested that improving indicators with low values usually takes less effort or costs less than improving indicators with high values.

### Use of experimental animals, and human participants

In this research, we did not perform experiments on humans or use human tissue samples.

## Results

### Climate change, country success and cities’ quality of life (C^3^S-QL) models

Our research involved correlation analysis of 32 country success indicators, 9 city quality of life indicators, and 14 quantitative and qualitative climate change indicators (Supplementary Tables S1–S3). The 169 nations analyzed were allocated to a control cluster and an experimental cluster (“Methods”, Supplementary Table S1). The experimental cluster, containing 15 metrics, was not examined using C^3^S models and maps. For the 169 countries, the study found a moderate median correlation (r = 0.579%) between 32 country success indicators (the control cluster of 17 metrics and the experimental cluster of 15 metrics), 12 climate change indicators, and two dimensions of the 2020 Inglehart–Welzel World Cultural Map^26^ (Table 1). Median strong correlations (in absolute terms) between the 238 cities (r = 0.747) and 169 countries (r = 0.963) are shown in Supplementary Table S3A,B. After concluding the relationship investigation, it was determined that average, positive, and statistically significant relationships occurred between control cluster and experimental cluster metrics (r = 0.650) and traditional versus secular-rational values (r = 0.800, p < 0.01). Average, positive, and statistically significant relationships were also found between 12 climate change indicators and control cluster (r = 0.505) and experimental cluster (r = 0.516) metrics, and the two dimensions of the 2020 Inglehart–Welzel Cultural Map of the World (r = 0.486) (Table 1 and Supplementary Table S3). The correlation outcomes validate the second hypothesis. All of the correlations have been calculated in absolute terms. The colors (green or pink) in Supplementary Table S3c,d indicate the spillover effect—synergies or trade-offs between country success and city quality of life to climate change indicators.

**Table 1:** Average correlations of the control and experimental clusters of the success indicators of 169 countries, two dimensions of the 2020 Inglehart–Welzel World Cultural Map^26^, and climate change metrics.

	12 climate change indicators	17 control cluster metrics used in the calculations of C^3^S models and to generate C^3^S maps	15 experimental cluster metrics not used in the calculations of C^3^S models and to generate C^3^S maps	2020 Inglehart–Welzel World Cultural Map^26^, two dimensions	Total average correlations
	1	2	3	4	5
12 climate change indicators	1	|0.505|	|0.516|	|0.486|	|0.448|
17 control cluster metrics		1	|0.650|	|0.635|	|0.607|
15 experimental cluster metrics			1	|0.699|	|0.679|
2020 Inglehart–Welzel World Cultural Map^26^, two dimensions				1	|0.584|
Total average correlations					1

Relationships between the 14 quantitative and qualitative climate change metrics and between the 15 country success and 9 city quality of life parameters have been established by the C^3^S and C^3^QL models. The relationships are statistically significant. The C^3^S models, a formal representation of the C^3^S maps, evaluate the links between the 15 independent control cluster variables and the 12 dependent climate change variables.

Linear regression revealed that changes in countries’ success values explain an average of 66.54% of the dispersion of each of the values of the climate change indicators used in the study (Table 2). The 15 country success control cluster variables for the 169 countries taken from the pool modeled in the twelve C^3^S models explain 95.4% of the dispersion related to the ND-GAIN index, 77.3% of the dispersion of the EPI for climate change, and 75.3% of the dispersion of the EF carbon footprint variables (Table 2). Table 2 shows how country success and its factors influence climate change indicators. As we analyzed the relative success increases in the 169 countries, we determined that nine climate change indicators have synergies, and three have trade-offs. Positive values show synergies between countries’ success and their indicators and climate change indicators, whereas negative values show trade-offs.

**Table 2:** How countries’ success and their factors influence climate change indicators.

Energy use rank	ND-GAIN (Notre Dame Global Adaptation Initiative) index	Public belief in the climate emergency	Country that says we should do everything necessary	EPI (Environmental Performance Index) climate change	EPI climate change rank	EPI greenhouse gas emissions per capita	Climate perceptions index	EF carbon footprint	CO_2_ emissions from fossil fuel combustion and cement production (tCO_2_/capita)	CO_2_ emissions embodied in imports (tCO_2_/capita)	Carbon pricing score at EUR60/tCO_2_
The Climate Change and Country Success (C^3^S) models*											
1	2	3	4	5	6	7	8	9	10	11	12
** −**	**+**	**+**	**+**	**+**	**−**	**−**	**+**	**−**	**−**	**−**	**+**
Percentage increase in the climate change indicator when a nation’s success increases by 1%											
− 0.608	0.493	0.156	0.241	0.533	− 0.952	− 0.927	0.099	0.861	1.503	2.564	0.447
Percentage of the dependent climate change variable under analysis (R^2^) explained by the independent variables of a nation’s success											
44.9	95.4	51.4	78.6	77.3	77.9	74.0	56.9	75.3	54.1	76.2	64.1

A positive (+) or negative (–) * sign specifies that a larger (smaller) climate change indicator value corresponds to a positive value for policy-makers.

To measure the effect that changes in each of the independent country success variables included in the model make on the dependent climate change variable, we need to calculate the elasticity coefficient for each independent variable. The coefficient indicates by what magnitude the value of the dependent climate change’ variable changes when the value of an independent variable goes up by 1%. Table 2 shows the percentage improvement of the climate change indicator value when the value of a country’s success indicator increases by 1%. A 1% increase in the human development index, global gender gap, and happiness index indicators, for instance, is followed by average increases of 0.93%, 0.903%, and 0.814%, respectively, in the 12 climate change indicators (Supplementary Table S4b).

Our research evaluates countries' success and sustainability elasticities to demonstrate a synergetic and trade-off character of nations' success and sustainability. Details of the synergies (marked green) and trade-offs (marked pink) between country success metrics and climate change indicators are presented in Supplementary Table S4b. Supplementary Table S4b displays, as a percentage, either improvement or deterioration (observed synergies and trade-offs) of climate change indicators when individual country success indicators improve by 1%. Supplementary Table S4b shows that a 1% improvement in country success indicators leads to the increase of 128 out of 180 climate change indicators (71%) and to the decrease of 52 indicators (29%). These numbers confirm that sustainability has a trade-off and synergetic character. So, we confirmed the nations' success and sustainability elasticity for climate change indicators.

We presented the determination coefficient (R^2^), which estimates what degree countries’ success metrics models predict climate change indicators in Supplementary Table S4c. Supplementary Table S4 confirms Hypothesis 1, which states that countries’ and cities’ climate change performance can be improved beyond traditional environmental, economic, and other conventional measures. Our research demonstrates that climate change can also be reduced by improving human development, ensuring gender equality, and improving democracy, as well as happiness indicators. C^3^QL, C^3^S, and the related integrated models support the holistic approach to climate change taken by Rogelj^15^, Fuldauer et al.^21^, and other researchers.

Supplementary Sect. 2 and Supplementary Tables S4–S8 present the descriptive statistics and effect sizes of the twelve C^3^S models. The third hypothesis has been proven by the investigation outcomes associated with the C^3^QL and C^3^S models, as well as the relationships recognized between the country, city, and climate change indicators (Supplementary Tables S3 and S4, Figs. 1, 2, 3). Our study proves Hypothesis 3, which demonstrates that improvements in the success of countries, the quality of life of cities, and their indicators are now mostly accompanied by improvements in climate change indicators.

Theoretical (Supplementary Table S9) and practical (Supplementary Table S4) MCDM and regression analyzes of the climate change policy alternatives of the 169 countries are presented in detail in the Method, Discussion, and Conclusions sections.

### Climate change, country success and cities’ quality of life (C^3^S-QL) maps

C^3^QL, C^3^S, and their integrated maps, which were created in this study and show a two-dimensional view, demonstrate how the success of the 169 countries and the quality of life of the 238 cities affect the improvement of climate change indicators. The x-axis indicates success and quality of life, and the y-axis indicates climate change metrics. Supplementary Tables S1 and S2 provide lists of all of these indicators, their values, and significance. This research looked at 12 national and 2 city-level climate change metrics represented along the y-axis. The criteria interaction, shown in Figs. 1, 2, 3 in the main paper and Supplementary Tables S1–S9, is statistically significant, implying that increased values of country success and city quality of life usually positively affect climate change indicators. Each country cluster has a tight outline and represents clear clustering.

The C^3^S ND-GAIN World Map (Fig. 1) presents a visualization of the 163 countries showing the relationship between a country’s success priority (x-axis) and ND-GAIN index (y-axis) (on the country and cluster level). Vulnerability and readiness are two key dimensions looked at in the ND-GAIN index^44^. Vulnerability refers to a country’s sensitivity, exposure, and ability to adapt to the negative effects of climate change; six life-supporting sectors (water, food, human habitat, ecosystem service, health, and infrastructure) are considered to measure a country’s vulnerability. Readiness measures a country’s ability to make use of investments toward its adaptation actions and looks at three components, which are social readiness, governance readiness, and economic readiness^44^. We applied the first five steps of the INVAR technique^33^ to determine the priority of each country (Supplementary Table S1). Where national data are deficient, the countries selected for the current study are not displayed. Country success is strongly negatively related to the ND-GAIN index (the relationship between these criteria is negative and statistically significant, r =  − 0.923, p > 0.01).

Six of the analyzed countries—Haiti, Luxembourg, Austria, Israel, Singapore, and China—do not fall into their clusters. China does not fall into its Confucian cluster, and Luxembourg and Austria do not fall into their Catholic Europe cluster. However, there are reasons (geographical proximity, tight economic and cultural ties, religious affinity, common and associated history, and analogous development levels) why these countries are outside of their clusters. In the case of Haiti, a Latin American country, most of its inhabitants belong to the ethnic group of Afro-Haitians, and so the country falls into the cluster of African-Islamic countries. China, which is from the Confucian cluster, falls between the African-Islamic cluster and the Orthodox cluster. The culture of Israel from the West and South Asia cluster is historically and politically more closely related to Europe. Singapore has a multicultural population and uses English as its lingua franca. The cultural identity of Singaporeans negotiates fluidly between two divergent orientations—those of the global citizen and the local Singaporean. More than half of the populations in Bosnia and Herzegovina (which is attributed to Orthodox Europe) and Indonesia and Malaysia (which are attributed to the West and South Asia cluster) is traditionally Muslim, and these countries therefore fall into the African-Islamic cluster in the map (Fig. 1).

The City Quality of Life and Global Gridded Model of Carbon Footprints^45^ World Map (Fig. 2) analyzes 155/238 cities from 60 countries (r = 0.6692) (Fig. 2). Several cities from the Confucian cluster—Hong Kong, Qingdao, Shandong, Shanghai, Beijing (CN), and Seoul (KR)—and the African-Islamic cluster—Jeddah (Jiddah) and Riyadh (SA), Dubai and Abu Dhabi (AE), and Doha (QA)—fall into clusters other than expected. Qingdao (CN), Shandong (CN), Shanghai (CN), Beijing (CN), and Seoul (KR) fall into the African-Islamic cluster. Hong Kong (HK) falls into the English-speaking and Protestant Europe cluster. The cities of Jeddah and Riyadh in Saudi Arabia, Dubai and Abu Dhabi in the UAE, and Doha in Qatar fall into the English-speaking and Protestant Europe cluster. Many factors contribute to this cluster assimilation. Hong Kong (CN), for instance, is in the English-speaking and Protestant Europe cluster for historical, cultural, and economic reasons. For the years between 1841 and 1997, except for the period of Japanese occupation between 1941 and 1945 during the Pacific War, Hong Kong was a British colony and later a British dependent territory. The other cities in China (Qingdao, Shandong, Shanghai, and Beijing) also have relationships with the African-Islamic cluster because China is Africa’s second-largest trading partner as a single country, and the leading infrastructure investor and lender on the continent^46^. Politically, the growth of relations between China and African nations has important meaning for both Africa and China, as well as across other emerging markets^46^.

Tel Aviv-Yafo (IL) and Singapore (SG) also do not fall into their West and South Asia clusters, falling instead into the English-speaking and Protestant Europe cluster. In the case of Israel, new Jewish immigrants from various European countries arrived in Palestine, bringing with them their own ideology and the dream of creating one identity, one culture, and one destiny for them all in their new destination^47^. Singapore, for its part, was colonized by the British in 1819 and gained independence in 1965^48^. For all its flaws, British rule laid the foundations for the remarkable economic success the city-state currently enjoys today^49^. Local ruling politicians also believe that, unlike Singapore, which has built on and improved the legacy left to it by the British, other former British colonies have squandered that legacy. These factors led to cluster assimilation and thus these countries fall into the English-speaking and Protestant Europe cluster (Fig. 2).

Figure 3 shows a snippet of the Integrated World Map of Climate Change, Country Success, and City Quality of Life. The country data points on the x-axis are the averages calculated based on the priority ranks of each country’s cities on the quality of life index and the country’s success priority rank (Supplementary Sect. 3). The country data points on the x-axis are the averages calculated based on the priority ranks of the country’s cities on the quality of life index and a country’s success priority rank. The UK, for instance, ranks 18th based on its success priority, while the average priority rank of its cities on the quality of life index is 17. The value for the UK on the x-axis, then, is 17.5. The correlation between the averages calculated based on the priority ranks of the country’s cities on the quality of life index and the country’s success priority rank is strong (r = 0.818). The y-axis shows the averages calculated based on the priority ranks of the country’s cities related to their footprint per capita (tCO_2_/cap) and the country’s priority rank on the ND-GAIN index. Let’s take the UK as an example again. The country ranks 11th based on its priority on the ND-GAIN index, while the average priority rank of its cities based on their footprint per capita (tCO_2_/cap) is 63. The value for the UK on the y-axis, then, is 37. The correlation between the averages calculated based on the priority ranks of a country’s cities related to their footprint per capita (tCO_2_/cap) and the country’s priority rank on the ND-GAIN index is positive and average (r = 0.576).

The *R*ecommendation m*e*thod for *c*limate *c*hange mitigation, *a*daptation and resilience *st*atistical and *m*ulticriteria analysis (the RECASTM method) developed as part of this research was used to analyze a number of alternatives to maximize country success in an integrated way and foster effective climate change resilience building, mitigation, and adaptation behaviors. Using the RECASTM method, we determined that GDP per capita in PPP (N_2_ = 100%), the environmental performance index (N_8_ = 97.7%), GDP per capita (N_1_ = 97.52%), the human development index (N_5_ = 90.78%), healthy life expectancy (N_13_ = 85.35%), the corruption perceptions index (N_4_ = 83.53%), and the global gender gap (N_6_ = 74.65%) were the success variables with the strongest impact on 12 climate change indicators in 169 countries (Supplementary Table S4).

## Discussion and conclusion

For decades, researchers have been debating the idea that climate change metrics, national indicators of success, and city quality of life indicators move in tandem^50–53^. This phenomenon reflects a big picture trend characteristic of our modern world. We used statistical and MCDM analyzes to measure how the success of 169 countries and the quality of life of 238 cities affect climate change indicators. One result of this research is the RECASTM method for the generation of potential climate change mitigation, adaptation and resilience alternatives, and rational option selection. The RECASTM method is described in detail below (Method).

A number of researchers have used a holistic approach^15, 21^ and multiple dimensions^16, 54^ of climate targets in their studies. Fuldauer et al.^21^ conceptualized 12 different chronic (gradual) and acute (extreme) climatic impact drivers, a holistic set of 22 socio-economic sectors and ecosystems, and the complex influences of bi-directional nature between all 169 SDG targets. A holistic understanding of how societal and individual reactions to climate change interact with economic, social, and biophysical processes is required in the design and implementation of climate adaptation actions^54^. In our study, we also looked at country success, city quality of life, and multiple related climate change dimensions from an integrated sustainability perspective, and the result is a view containing a broad spectrum of synergies and trade-offs intertwined. The social benefits and adverse effects of carbon pricing (x_12_) analyzed in our research, for instance, have been thoroughly examined by the Climate Action Network^55^. The indicators of country success, city quality of life, and related statistical and subjective-survey climate change metrics along with C^3^QL, C^3^S, and their integrated maps and models used in our research showcase an integrated sustainability approach to climate change.

This research differs from other existing studies in one key aspect: our analysis took an integrated sustainability approach at the way country-level economic, social, political, cultural, and environmental context affect changes in climate change indicators, whereas other researchers have focused on only a few of these indicators at a time.

Yet global research supports our findings in several directions. Our research, for instance, has shown synergistic effect between country success and climate change indicators and demonstrated that increasing country success led to a positive impact in 9 of the 12 climate change indicators (Table 2). We analyse seven social (27 trade-offs, 57 synergies), four economic (13 trade-offs, 35 synergies), one environmental (3 trade-offs, 9 synergies), and three political (9 trade-offs, 27 synergies) indicators across 169 countries, indicating synergies with climate change indicators at 68%, 73%, 75%, and 75% respectively. Overall, 71% of climate change indicators improved (52 trade-offs, 128 synergies; see Supplementary Table S4b).

Our findings are in agreement with the priors studies, which analyzed individual country success indicators such as the use of non-fossil energy and total factor productivity^58^, non-corruptive practices^59^, ecological effects of green taxes in the transportation sector^60^, technological advancements, green energy consumption, energy efficiency^61^, digital trade and renewable energy consumption^62^, and technological innovations^63^. The findings presented by researchers^60^, who analyzed the phenomenon known as the Environmental Kuznets Curve, showed that higher economic growth leads to long-term climate change recovery.

The goal behind C^3^QL, C^3^S, and their integrated maps is a complex analysis of how climate change indicators, country success, and city quality of life interconnect. Mapping these elements allows the relationships to be illustrated. These maps and models show spillover effects between climate change indicators and country success and city quality of life and demonstrate synergies or trade-offs of climate change indicators when the country's success or the quality of life has higher or lower values. Various other studies^64, 65^ present similar findings, pointing to the need for continuous improvement in the sustainability of cities and countries.

The comparative values of a country’s success and its climate change indicators, therefore, were not much affected by changes in the number of countries we analyzed or in their climate change and success indicator sets. Nor did the choice of climate change and success indicators and their systems have a significant impact on each country’s eventual location within the group of seven country clusters used in this research. This, then, has validated the second hypothesis that country success, city quality of life, and their system of indicators and climate change metrics are interdependent, and this fact is visually reflected in C^3^S, C^3^QL, and the integrated maps. Figures 1, 2, 3, for instance, illustrate that countries that rank higher concerning with success metrics and cities that rank higher concerning with quality of life metrics figures have higher climate change indicators value, and the figures therefore visually confirm the second hypothesis. These outcomes of our study coincide with findings of other researchers^66^.

Looking at the seven cultural clusters, we see stable similarities related to contextual factors—national success and city quality of life, because the direction of success and quality of life is generally similar in cultures that share common traits. Higher-level country success metrics usually translate into synergies in quality of life and climate change indicators, as indicated by the average correlations determined in our research. The general median of all correlation coefficients between the climate change indicators and country success is moderate (r = 0.557), as seen in Table 1. Many indicators of a city’s quality of life, a nation’s success, and climate change are intertwined and move together, so they cannot be changed in isolation. These findings are confirmed by Sobel and Coyne^67^ and Coyne and Sobel^68^.

Our research determined a moderate median correlation (r = 0.491) between statistical and survey-based climate change indicators. Jones and D’Errico^34^ produced similar research findings with a moderate correlation between the data from objective and subjective approaches. The growing supply of suitable data and the increasing body of knowledge generated in studies around the world sustains continuous improvement of the C^3^QL, C^3^S, and integrated models, which thus show an increasingly accurate and realist picture and can be an efficient policymaking tool.

Practical conclusions and digital recommendations have been derived following MCDM analysis, statistical calculations, and the use of C^3^S and C^3^QL maps and models (“Methods”, “Results”). Among them is our finding that higher country success or city quality of life is usually accompanied by higher climate change metrics. This research shows that a country or city responds to climate change challenges depends on its macro level context and existing quality of life and success indicators level. This information should be considered before specific policies are established. Yet some countries fail to do this in a consistent manner. Possible multiple-criteria scenarios were generated and analyzed to achieve balancing goals and the lowest possible adverse effect on the well-being of countries and cities, yet at the same time ensure the highest possible climate change reduction through advanced climate mitigation, adaptation, and resilience actions.

From an integrated sustainability perspective, the RECASTM method discussed in our case study is a tool that helps to determine the most efficient ways to achieve customized improvements in the climate change indicators of specific countries (“Methods”). Twelve Climate Change and Country Success (C^3^S) models were created to analyse seven social, four economic, one environmental, and three political country success indicators. The countries’ average utility (competitiveness) degree in the models was 71.74% for social factors, 80.62% for economic factors, 97.7% for environmental factors, and 71.59% for political factors. Other researchers have expressed similar opinions^56, 57^. Our research direction was very similar and the findings confirm the points stated below. Supplementary Table S4 and global research have shown that some areas need to be explored more, such as better gender equality^69^, corruption^70^ reduction, emphasis on human development^71^, advances in democracy^72^, and happiness^73^. Such research emphasizes social^74, 75^ and cultural^76, 77^ factors, as well as supporting our regression and MCDM analysis results, which prove our first hypothesis that improvements in climate change indicators and mitigation, adaptation, and resilience actions can be achieved for countries and cities considered without sticking just to environmental, economic, and other traditional means (Supplementary Table S4).

Best practices^41–43^ can be taken into account to perform multi-variant options design and MCDM analysis of country- and city-level climate change mitigation, adaptation, and resilience policy alternatives, followed by the identification of rational decisions. We must also assess these options in a local context compared to a metrics system related to the local context. Once combinations of rational options are established, an appropriate response to the real-world situation, which is constantly shifting, can then be ensured. Most cities and countries need consistent policies and fast action to achieve better control and improve their climate change indicators in line with the best global practices. These findings can assist as useful strategies and can also act as a scorecard for cities and countries in their efforts to operate more successfully toward a sustainable future.

In the opinion of Stiglitz^64^, higher per capita incomes usually go together with higher national social indicators. A common agenda for governments in low-income countries is to put economic development first and protection of the environment second. In countries with incomes in the lower-middle or upper-middle ranges, the primary task involves the transformation of extensive economic growth paradigms into intensive ones. In the case of high-income countries, governments should make a point of vigorously promoting and advocating cleaner, environmentally friendly lifestyles and production patterns. However, residents in countries with higher incomes have greater demands as far as living standards are concerned, and satisfaction of their needs often requires greater energy consumption^65^. This means that policymakers must encourage these residents to develop living habits that are better for the environment and that are marked by lower consumption levels, if air quality is to be improved. The research findings discussed in this article are many confirmed by prior studies^64, 65^. The opinion these researchers express is consistent with the validation of our third hypothesis related to C^3^S, C^3^QL, and the integrated models and maps that the increasing success of a country and improvement of its city quality of life is mostly accompanied by an improvement in climate change indicators.

This investigation naturally has some limitations and weaknesses, and thus can be improved further. The aspects that require supplementary reflection in this field are listed below. In total, 169 and 173 countries were analyzed in this research, but by adding even more countries over a more extended time period of analysis, the accuracy of the C^3^S maps and models would improve and the global situation would be better reflected. However, the use of a large number of indicators in a system would mean that fewer countries would incorporate all of the relevant indicators. All of the indicators discussed in this study had the same weight. One future plan is the further evaluation of the significance of the indicators to make the C^3^S maps and models more accurate. For this purpose, an integrated approach will be taken, with both objective and subjective methodologies applied, and the significance of the indicators will be recalculated. This research applied a multiple linear regression model. To present more accurate descriptions of the existing situation, future C^3^S maps and models will be compiled by applying machine learning and data mining, as well as robust, stepwise, nonlinear, and nonparametric regression methods.

This study looked at all indicators on a national level. Different regions in a single country can be developed to different levels, however, and multicultural countries and cities also have diverse cultural and ethnic communities. Within a single country, indicators for different regions should therefore be assessed differently. We therefore expect future C^3^S maps and models to be based on specific systems of criteria with different values and significances when developed for specific multicultural countries. The basis of the 2020 Inglehart–Welzel Cultural Map of the World is the European Values Survey and the World Values Survey^26^, while the C^3^S maps are based on statistical indicators. However, these maps correlate significantly with one another (Supplementary Table S3). In the future, we expect that surveys performed at the national level and broader statistical indicators will be examined to establish how they are linked by dependencies. Investigation of other aspects of the three dimensions of sustainability (e.g., markets, global links, economy, people, inequality, and poverty) is planned for the future. Other features of the INVAR technique^33^, not used in the current study, will be applied to make the big picture of the C^3^S more comprehensive. The expected outcome is an extensive set of more efficient and more appropriate recommendations applicable in C^3^S maps and models.

## Supplementary Information

Supplementary Information 1.

Supplementary Information 2.

## Data Availability

Our research relies on data from multiple sources. All the original datasets and raw data used in this study are publicly available from open sources. All sources and source links for countries’ success (control and experimental clusters), climate change, and Inglehart–Welzel cultural map of the world indicators used in this study are listed in Supplementary Table S1. All sources and source links for cities’ quality of life and climate change indicators are listed in Supplementary Table S2. The global data used for comparison are from the United Nations Development Programme, the UN Sustainable Development Solutions Network, the UN Office for Disaster Risk Reduction, the Sustainable Development Solutions Network, the World Economic Forum, the World Bank, TheGlobalEconomy.com, the Economist Intelligence Unit, the Socioeconomic Data and Applications Center, the World Health Organization, the Freedom House, the Heritage Foundation, Transparency International, Credit Suisse Group AG, the Fund for Peace, the Institute for Economics and Peace, World Values Survey Association, SolAbility, the Our World in Data, the Global Footprint Network, the Yale Center for Environmental Law & Policy, Numbeo, Meta, C40 Cities Climate Leadership Group (C40 Knowledge Hub), and the Gridded Carbon Footprint dataset, as well as global and national statistics and publications. The author (Arturas Kaklauskas) can deliver the applied raw data used for obtaining the conclusions in this paper to others upon request. All the data generated in this study are provided in the main paper and the Supplementary Information.
