# Supplementary material for: Synergy of climate change with country success and city quality of life
Source: Sci Rep. 2023 May 15;13:7872. doi: 10.1038/s41598-023-35133-4 (PMC10184970; doi:10.1038/s41598-023-35133-4)
Supplement: Supplementary file 2 — Supplementary Information 2. [file 41598_2023_35133_MOESM2_ESM.docx]

Supplementary Materials for

**SYNERGY OF CLIMATE CHANGE WITH COUNTRY SUCCESS AND CITY QUALITY OF LIFE**

Arturas Kaklauskas *et al.*

*Corresponding author. Email: [arturas.kaklauskas@vilniustech.lt](mailto:arturas.kaklauskas@vilniustech.lt)

**This PDF file includes:**

Supplementary Text

Tables S1 to S9

**Supplementary Text**

**1. Climate change mitigation, adaptation and resilience**

Climate change mitigation, adaptation, and resilience actions include a wide range of alternatives discussed briefly below. Climate change mitigation actions aim to curb climate change by achieving lower greenhouse gas emissions in the future or removing from the atmosphere those gases that have already been emitted^1^. Mitigation efforts can make emissions lower by energy conservation, better efficiency, and shift to sustainable energy sources^2^. Agroforestry, together with sustainable land and soil management, is one of the nature-based solutions that can help to cut carbon emissions and, thus, reduce the effects of climate change^3^. O’Neill et al.^4^ have suggested that adaptation actions can be grouped into institutional, behavioral, and cultural options; technological options; infrastructural options; and nature-based options. These four categories offer people protection by making them more capable of adapting to climate change^3^. Climate resilience means that the social environment, economic environment, and ecosystems can cope with a hazardous disturbance, trend, or event  by reorganizing or responding to such threats in ways that keep their essential identity and function intact, yet make them equipped with a capacity to adapt, learn, and transform^5^. One of the main directions in climate resilience, as von Braun et al.^3^ have suggested, is lifestyle reform in wealthy nations, where populations must reduce consumption and be more aware of their impact on the environment.

**2. Descriptive statistics and effect sizes of the models**

Descriptive statistics of the 12 C^3^S models are presented in Table S5. The minimum (lowest) and the maximum (highest) values of a variable in the set of values the variable in question can take show the interval within which its values vary. The mean is the average value of the full set of values a variable can take, usually equal to the arithmetical average. The standard deviation shows the distribution of a variable’s values around the mean. Kurtosis shows whether or not most values are densely clustered around the average. Skewness shows whether or not values are symmetrically distributed around the mean. In case of kurtosis and skewness, values between −3 and 3 for skewness and between −10 and 10 for kurtosis are deemed acceptable. If skewness is around 0 and kurtosis is around 3, the allocation of the values of the variable in question within the value set in question follows the law of normal distribution.

Table S6 presents the results for the goodness-of-fit testing of C^3^QL and C^3^S models. The determination coefficient R^2^ measures the accuracy of a C^3^S model in predicting a country’s climate change indicators. The model’s dependent climate change variables represent the outcome. The closer a C^3^S model’s R² is to 1, the more accurate the model. The determination coefficient (R^2^), hence, shows the share of the dependent sustainability variable’s variation that can be predicted by looking at the independent success variables. R^2^ thus shows what share of the variation in the dependent climate change variable can be predicted by examining the independent control cluster variables. R^2^ is used in the context of the C^3^S models designed to predict the success and sustainability countries will achieve in the future and to test the second and third hypotheses (Table S6). Beta weights can be arranged according to rank to ascertain the best predictor variable in a multiple linear regression (Table S7).

An effect size in statistics shows whether the relationship between two variables is strong or weak, or an estimate of that quantity based on the sample^6^. The coefficient of regression in a regression, the relationship between two variables, the mean difference, or the risk that a particular event will happen are examples of effect sizes^7^. We have followed the guidelines for calculating, reporting, and interpreting effect sizes developed by Durlak^8^. The guidelines recommend that effect sizes are used in studies, as well as how to calculate and interpret them. In statistics, effect size measures the strength of the relationship between two variables or is used to calculate an estimate of that amount as a sample (*67*). Effect sizes can be the mean difference, the correlation between two variables, the regression coefficient in a regression, or the risk that a specific event will occur (*68*). This research includes five measures of effect size—the coefficient of determination (R²), Pearson’s correlation coefficient (r), a standardized beta coefficient (β), standard deviation, and p-values—as they can be calculated from almost any study design and their calculation is required for meta-analysis. These five measures were extracted from all studies where available.

The degrees of impact calculated for the C^3^QL dependent climate change variables show that changes in the values of city quality of life indicators such as the purchasing power index (If=6.971), quality of life index (If=6.672), cost of living index (If=4.727), and pollution index (If=3.253) have the biggest impact on both indicators. These city quality of life indicators have degrees of impact higher than the average impact indicator values (M=3.229; SD=0.792) (Table S8). Tables S4 and Table S8 show that success and quality of life are significant predictors of which climate change policies a country should prefer. For example, a few cases were observed in which a country’s success (unemployment rate) and a city’s quality of life (safety index) indicators had no noticeable impact on climate change.

Table S4c shows how climate change indicators depend on the coefficients of determination (R²) of the regression equations for country success indicators; the coefficients show the share (percentage) of the dispersion of the values of a specific climate change indicator explained by changes in the values of a respective country success indicator. The values of this coefficient, therefore, show to what extent a country success indicator can be used to forecast a respective climate change indicator. The average of the coefficient of determination of a respective country success indicator with respect to climate change indicators shows the extent to which the specific country success indicator can be used to forecast all climate change indicators. The environmental performance index, GDP per capita in PPP, human development index, and fragile state index, for instance, explain, on average, 43%, 41.6%, 39.1%, and 38.3%, respectively, of the 12 climate change indicators. These should, therefore, be used to forecast climate change indicators. Other country success indicators explain the 12 climate change indicators to a lesser extent (Table S4c).

The degrees of impact calculated for the C^3^S climate change dependent variables show that the most significant integrated impact on all climate change indicators is made by changes in the values of indicators such as the environmental performance index (If=43,495), GDP per capita in PPP (If=40,133), the human development index (If=37,305), the fragile state index (If=31,802), healthy life expectancy (If=31,429), GDP per capita (If=29,194), and the happiness index (If=29,044). The values of these country success indicators show a higher degree of impact than the average indicator impact values (M=28,739; SD=2,166). The C^3^QL environmental performance index has the most significant impact on climate change variables (Table S4a).

**3. The INVAR method**

The second step involved data collection and multicriteria study of 169 nations and 238 cities by means of the INVAR (Degree of Project Utility and Investment Value Assessments) method (Method, Tables S1 and S2). The references to well-known databases, publications, and other resources used in this research are listed at the bottom of Tables S1 and S2, which list the countries and cities analyzed in this article and present the indicator sets in detail. The success and competitiveness of the cities and countries examined in this study can only be considered using a system of indicators with various different dimensions. Some countries were excluded from this analysis, because no official comparable data were available for them. The INVAR method offers a fairly easy way to assess and then identify the most competitive countries and cities with the highest quality of life. The same method was used to create a generalized (reduced) indicator Q_j_ that depends directly and proportionately on the relative influence of the weights q_i_ and values x_ij_ of the indicator being compared on the end result. The success, Q_j_, of a city/country a_j_ shows its performance related to quality of life and success indicators. The cities with the highest quality of life and the most successful country will always achieve the biggest value Q_max_. The success of all other cities/countries a_j_ will always be lower than Q_max_. This means that their performance in relation to quality of life and success will be below that of the most successful city/country. To assess the pluses and minuses of the performance achieved by these cities/countries more effectively, and to compare them with the needs and goals of politicians and other stakeholder groups, each city’s/country’s quality of life and degree of competitiveness has to be determined. This degree depends directly on the system of metrics. If one city/country performs better on the economic indicators, another city/country performs better on the social, environmental, and political metrics, and if MCDM determines identical levels of success for both cities/countries, the degree of the quality of life and competitiveness of the two cities/countries will also be identical. As a city’s quality of life and a country’s success increases, so does its competitiveness, and vice versa. This means that all cities and countries in question will have degrees of quality of life and competitiveness between 0 percent (lowest value) and 100 percent (highest score) (i.e. all measurements of anything going to be between 0% and 100%). This will make it easier to assess the cities’ quality of life and the countries’ competitiveness quantitatively.

**References**

1. IPCC, 2021: Annex VII: Glossary [Matthews, J.B.R., V. Möller, R. van Diemen, J.S. Fuglestvedt, V. Masson-Delmotte, C. Méndez, S. Semenov, A. Reisinger (eds.)]. in *Climate Change 2021: The Physical Science Basis. Contribution of Working Group I to the Sixth Assessment Report of the Intergovernmental Panel on Climate Change* 2215–2256 (Cambridge University Press, Cambridge, United Kingdom and New York, NY, USA, 2021).

2. Olivier, J. *TRENDS IN GLOBAL CO AND TOTAL GREENHOUSE GAS EMISSIONS - 2021 Summary Report*. (Netherlands Environmental Assessment Agency. Retrieved from https://policycommons.net/artifacts/3151857/trends-in-global-co-and-total-greenhouse-gas-emissions/3949682/ on 24 Jan 2023. CID: 20.500.12592/00zkbz, 2022).

3. von Braun, J., Ramanathan, V. & Turkson, P. K. A. Climate mitigation is not enough — focus on resilience now. *Nature* **610**, 257 (2022).

4. O’Neill, B. *et al.* Key Risks Across Sectors and Regions. in *Climate Change 2022: Impacts, Adaptation and Vulnerability. Contribution of Working Group II to the Sixth Assessment Report of the Intergovernmental Panel on Climate Change* 2411–2538 (Cambridge University Press, Cambridge, UK and New York, NY, USA, 2022).

5. IPCC, 2022: Summary for Policymakers. in *Climate Change 2022: Impacts, Adaptation and Vulnerability. Contribution of Working Group II to the Sixth Assessment Report of the Intergovernmental Panel on Climate Change* (eds. Pörtner, H.-O. et al.) 3–33 (Cambridge University Press, Cambridge, UK and New York, NY, USA, 2022).

6. Kelley, K. & Preacher, K. J. On effect size. *Psychol. Methods* **17**, 137–152 (2012).

7. Wilkinson, L. & Task Force on Statistical Inference, American Psychological Association, Science Directorate. Statistical methods in psychology journals: Guidelines and explanations. *Am. Psychol.* **54**, 594–604 (1999).

8. Durlak, J. A. How to Select, Calculate, and Interpret Effect Sizes. *J. Pediatr. Psychol.* **34**, 917–928 (2009).

9. Haerpfer, C. *et al.* World Values Survey: Round Seven - Country-Pooled Datafile Version 4.0. (2022) doi:10.14281/18241.18.

TABLES

**Table S1. Success, climate change, and the two-dimensional 2020 Inglehart–Welzel World Cultural Map^9^ for 169 countries: original data and multiple criteria analysis results.**

**Table S2. Quality of life and climate change indicators for 238 cities.**

**Table S3. Correlations of (A) 169 countries’ success, (B) 238 cities’ quality of life and climate change indicators, (C) country and (D) city indicators.**

Table S4. Practical multiple criteria and regression analysis of the climate change policy alternatives of 169 countries.

|  | | |  | **Control cluster: Country success indicators** | | | | | | | | | | | | | | |
| --- | --- | --- | --- | --- | --- | --- | --- | --- | --- | --- | --- | --- | --- | --- | --- | --- | --- | --- |
| **Climate change indicators** |  | | **Indicators' weight** | **GDP per capita, a_1_** | **GDP per capita in PPP, a_2_** | **Ease of doing business ranking, a_3_** | **Corruption perceptions index, a_4_** | **Human development index, a_5_** | **Global gender gap, a_6_** | **Happiness index, a_7_** | **Environmental performance index, a_8_** | **Freedom and control, a_9_** | **Economic freedom, a_10_** | **Democracy index, a_11_** | **Unemployment rate, a_12_** | **Healthy life expectancy, a_13_** | **Fragile state index, a_14_** | **Economic decline index, a_15_** |
|  | **Measuring units** | |  | International dollar | International dollar | Rank | Index | Index | Index | Index | Index | Index | Index | Index | % of total labor force | Years | Index | Index |
| Plus (+) or minus (-) indicates that a greater (lesser) criterion value corresponds to a greater significance by country: | | |  | **+** | **+** | **-** | **+** | **+** | **+** | **+** | **+** | **-** | **+** | **+** | **-** | **+** | **-** | **-** |
| **(a) The weights of the impact on twelve C^3^S regression models calculated for the dependent climate change variables (If)** | | | | | | | | | | | | | | | | | | |
| Energy use rank | kg of oil equivalent per capita | **+** | 1 | 2.124 | 3.682 | 3.656 | 1.597 | 3.957 | 2 | 1.781 | 5.659 | 0.424 | 1.968 | 0.732 | 0.841 | 1.423 | 1.71 | 1.796 |
| ND-GAIN index | score | **+** | 1 | 3.247 | 3.309 | 4.842 | 3.967 | 4.748 | 2.831 | 3.333 | 3.546 | 2.559 | 3.305 | 2.859 | 0.235 | 3.664 | 4.299 | 3.159 |
| Public belief in the climate emergency | % of population | **+** | 1 | 2.136 | 2.018 | 1.584 | 3.277 | 2.539 | 1.425 | 2.115 | 5.255 | 1.791 | 3.656 | 1.788 | 0.666 | 1.931 | 2.219 | 1.178 |
| Country who say we should do everything necessary | Proportion of respondents by country | **+** | 1 | 2.21 | 3.294 | 1.373 | 2.548 | 3.068 | 2.557 | 2.615 | 3.503 | 4.327 | 2.279 | 2.675 | 1.59 | 2.998 | 3.183 | 1.412 |
| EPI climate change | score | **+** | 1 | 2.326 | 2.464 | 2.675 | 2.628 | 3.18 | 2.766 | 2.588 | 5.474 | 2.059 | 2.554 | 2.531 | 0.498 | 3.173 | 2.863 | 2.213 |
| EPI climate change rank | rank | **+** | 1 | 2.216 | 2.337 | 2.647 | 2.653 | 3.27 | 2.735 | 2.596 | 5.468 | 2.192 | 2.542 | 2.506 | 0.519 | 3.191 | 2.925 | 2.424 |
| EPI greenhouse gas emissions per capita | score | **+** | 1 | 3.236 | 4.981 | 2.657 | 2.605 | 4.224 | 1.267 | 3.139 | 3.282 | 0.965 | 3.114 | 2.077 | 0.33 | 3.324 | 3.34 | 3.229 |
| Climate perceptions index | score | **+** | 1 | 0.867 | 0.66 | 1.215 | 1.018 | 1.044 | 4.394 | 1.315 | 1.204 | 4.254 | 0.914 | 3.271 | 0.213 | 2.095 | 1.961 | 1.545 |
| EF carbon footprint | score | **+** | 1 | 3.033 | 5.174 | 2.256 | 2.572 | 3.154 | 0.889 | 2.77 | 2.793 | 0.624 | 2.665 | 0.957 | 0.14 | 3.005 | 3.048 | 2.979 |
| CO₂ emissions from fossil fuel combustion and cement production | tCO2/capita | **+** | 1 | 2.046 | 4.101 | 1.144 | 1.357 | 2.593 | 0.613 | 1.828 | 1.601 | 0.609 | 1.81 | 1.031 | 0.593 | 1.79 | 2.051 | 3.419 |
| CO₂ emissions embodied in imports | tCO₂/capita | **+** | 1 | 2.5 | 4.446 | 1.539 | 2.722 | 3.162 | 2.231 | 2.431 | 1.821 | 1.32 | 2.469 | 1.748 | 0.597 | 1.734 | 2.496 | 1.936 |
| Carbon pricing score at EUR60/tCO₂ | % | **+** | 1 | 3.253 | 3.666 | 0.629 | 1.747 | 2.365 | 3.289 | 2.533 | 3.887 | 1.443 | 0.089 | 1.489 | 0.949 | 3.101 | 1.706 | 0.719 |
|  | | | | The degrees of impact calculated for the C^3^S climate change dependent variables show that the most significant integrated impact on all climate change indicators is made by changes in the values of indicators (these aggregate explanatory indicators are not used in the calculations) | | | | | | | | | | | | | | |
|  |  |  |  | **29,194** | **40,133** | 26,217 | 28,690 | **37,305** | 26,997 | **29,044** | **43,495** | 22,566 | 27,365 | 23,662 | 7,172 | **31,429** | **31,802** | 26,008 |
| **(b) The percentage increase of a climate change indicator value caused by a 1% increase in the country’s success indicator (P_id_)** | | | | | | | | | | | | | | | | | | |
| Energy use rank | kg of oil equivalent per capita | **-** | 1 | -0.150 | -0.260 | 0.445 | -0.444 | -1.203 | -1.009 | -0.710 | -0.841 | 0.077 | -1.038 | -0.216 | 0.136 | -0.662 | 0.543 | 0.552 |
| ND-GAIN index | score | **+** | 1 | 0.127 | 0.183 | -0.312 | 0.451 | 0.982 | 1.311 | 0.872 | 0.579 | -0.239 | 0.953 | 0.398 | 0.003 | 1.485 | -0.545 | -0.505 |
| Public belief in the climate emergency | % of population | **+** | 1 | 0.050 | 0.075 | -0.096 | 0.172 | 0.380 | 0.347 | 0.271 | 0.208 | -0.098 | 0.815 | 0.157 | 0.018 | 0.549 | -0.161 | -0.105 |
| Country who say we should do everything necessary | Proportion of respondents by country | **+** | 1 | 0.078 | 0.125 | -0.116 | 0.248 | 0.666 | 0.833 | 0.552 | 0.307 | -0.194 | 0.522 | 0.292 | 0.039 | 0.952 | -0.281 | -0.208 |
| EPI climate change | score | **+** | 1 | 0.138 | 0.190 | -0.344 | 0.440 | 1.133 | 1.700 | 0.853 | 0.825 | -0.032 | 1.023 | 0.493 | 0.104 | 1.742 | -0.579 | -0.138 |
| EPI climate change rank | rank | **-** | 1 | -0.166 | -0.246 | 0.615 | -0.782 | -2.069 | -3.024 | -1.520 | -1.480 | 0.544 | -1.828 | -0.879 | -0.194 | -3.159 | 1.090 | 0.811 |
| EPI greenhouse gas emissions per capita | score | **-** | 1 | -0.288 | -0.425 | 0.575 | -0.766 | -2.146 | -2.026 | -1.621 | -1.172 | 0.328 | -1.834 | -0.613 | -0.068 | -2.914 | 1.072 | 1.064 |
| Climate perceptions index | score | **+** | 1 | 0.020 | 0.026 | -0.082 | 0.095 | 0.193 | 0.868 | 0.265 | 0.133 | -0.163 | 0.155 | 0.218 | -0.017 | 0.584 | -0.154 | -0.134 |
| EF carbon footprint | score | **-** | 1 | 0.575 | 0.898 | -0.945 | 1.539 | 3.481 | 2.597 | 2.888 | 1.950 | -0.588 | 3.114 | 0.980 | -0.069 | 5.173 | -1.931 | -1.966 |
| CO₂ emissions from fossil fuel combustion and cement production | tCO2/capita | **-** | 1 | 0.462 | 0.790 | -0.876 | 1.126 | 3.252 | 1.281 | 2.676 | 1.457 | -0.281 | 2.643 | 0.573 | -0.150 | 4.014 | -1.642 | -1.811 |
| CO₂ emissions embodied in imports | tCO₂/capita | **-** | 1 | 0.744 | 1.081 | -1.265 | 2.252 | 4.192 | 6.429 | 4.238 | 2.647 | -1.093 | 4.715 | 1.720 | -0.256 | 7.813 | -2.578 | -2.552 |
| Carbon pricing score at EUR60/tCO₂ | % | **+** | 1 | 0.113 | 0.210 | 0.068 | 0.296 | 2.301 | 1.530 | 1.005 | 0.955 | -0.415 | 0.025 | 0.643 | -0.147 | 4.419 | -0.538 | -0.336 |
| **(c) The share of the dispersion of the values of a specific climate change indicator (R²) explained by changes in the values of a respective country success indicator** | | | | | | | | | | | | | | | | | | |
| Energy use rank | kg of oil equivalent per capita | **+** | 1 | 0.137 | 0.19 | 0.25 | 0.103 | 0.173 | 0.21 | 0.067 | 0.227 | 0.005 | 0.092 | 0.021 | 0.023 | 0.015 | 0.115 | 0.119 |
| ND-GAIN index | score | **+** | 1 | 0.604 | 0.674 | 0.713 | 0.755 | 0.819 | 0.255 | 0.614 | 0.767 | 0.376 | 0.552 | 0.496 | 0 | 0.526 | 0.826 | 0.703 |
| Public belief in the climate emergency | % of population | **+** | 1 | 0.309 | 0.289 | 0.175 | 0.375 | 0.242 | 0.084 | 0.169 | 0.379 | 0.221 | 0.264 | 0.223 | 0.012 | 0.161 | 0.247 | 0.102 |
| Country who say we should do everything necessary | Proportion of respondents by country | **+** | 1 | 0.481 | 0.511 | 0.163 | 0.507 | 0.48 | 0.311 | 0.453 | 0.532 | 0.561 | 0.31 | 0.498 | 0.036 | 0.311 | 0.489 | 0.258 |
| EPI climate change | score | **+** | 1 | 0.31 | 0.317 | 0.367 | 0.302 | 0.472 | 0.19 | 0.294 | 0.688 | 0.255 | 0.231 | 0.317 | 0.039 | 0.308 | 0.425 | 0.236 |
| EPI climate change rank | rank | **+** | 1 | 0.314 | 0.333 | 0.366 | 0.299 | 0.492 | 0.179 | 0.292 | 0.692 | 0.256 | 0.231 | 0.314 | 0.043 | 0.317 | 0.436 | 0.24 |
| EPI greenhouse gas emissions per capita | score | **+** | 1 | 0.384 | 0.543 | 0.368 | 0.33 | 0.609 | 0.092 | 0.383 | 0.499 | 0.107 | 0.267 | 0.176 | 0.006 | 0.31 | 0.484 | 0.476 |
| Climate perceptions index | score | **+** | 1 | 0.042 | 0.035 | 0.102 | 0.089 | 0.068 | 0.377 | 0.127 | 0.1 | 0.364 | 0.028 | 0.318 | 0.005 | 0.149 | 0.168 | 0.119 |
| EF carbon footprint | score | **+** | 1 | 0.534 | 0.692 | 0.282 | 0.36 | 0.446 | 0.041 | 0.332 | 0.365 | 0.094 | 0.294 | 0.124 | 0.002 | 0.263 | 0.443 | 0.469 |
| CO₂ emissions from fossil fuel combustion and cement production | tCO2/capita | **+** | 1 | 0.236 | 0.374 | 0.167 | 0.14 | 0.269 | 0.007 | 0.198 | 0.146 | 0.016 | 0.128 | 0.031 | 0.006 | 0.114 | 0.224 | 0.298 |
| CO₂ emissions embodied in imports | tCO₂/capita | **+** | 1 | 0.639 | 0.716 | 0.344 | 0.556 | 0.438 | 0.183 | 0.428 | 0.465 | 0.224 | 0.401 | 0.271 | 0.014 | 0.325 | 0.556 | 0.525 |
| Carbon pricing score at EUR60/tCO₂ | % | **+** | 1 | 0.31 | 0.314 | 0.002 | 0.09 | 0.185 | 0.109 | 0.134 | 0.301 | 0.113 | 0 | 0.134 | 0.037 | 0.215 | 0.185 | 0.053 |
|  | | | | The average of the coefficient of determination (R²) of a respective country success indicator with respect to climate change indicators shows to what extent the specific country success indicator can be used to forecast all climate indicators (these aggregate explanatory indicators are not used in the current calculations) | | | | | | | | | | | | | | |
|  |  |  |  | **35.8%** | **41.6%** | **27.5%** | **032.6%** | **39.1%** | **17%** | **29.1%** | **43%** | **21.6%** | **23.3%** | **24.4%** | **1.9%** | **25.1%** | **38.3%** | **30%** |
| **Country Success (Q_i_)*** | | | | **3.1601** | **3.2404** | **1.842** | **2.7067** | **2.9415** | **2.4188** | **2.2876** | **3.1657** | **1.5316** | **2.282** | **2.2997** | **0.8533** | **2.7657** | **2.2986** | **2.2069** |
| **Country Priority (P_i_)*** | | | | **3** | **1** | **13** | **6** | **4** | **7** | **10** | **2** | **14** | **11** | **8** | **15** | **5** | **9** | **12** |
| **Country competitiveness degree (N_i_), %*** | | | | **97.52%** | **100%** | **56.85%** | **83.53%** | **90.78%** | **74.65%** | **70.6%** | **97.7%** | **47.27%** | **70.42%** | **70.97%** | **26.33%** | **85.35%** | **70.94%** | **68.11%** |

* Link for calculated data: <http://iti4.vgtu.lt/Savas/daugkrit.aspx?sistemid=2161>

Table S5. Descriptive statistics of the 12 C^3^S models.

| **Descriptive statistics** | **Descriptive statistics of 12 C^3^S Models** | | | | | | | | | | | |
| --- | --- | --- | --- | --- | --- | --- | --- | --- | --- | --- | --- | --- |
|  | Energy use rank | ND-GAIN index | Public belief in the climate emergency | Country that says we should do everything necessary | EPI climate change | EPI climate change rank | EPI greenhouse gas emissions per capita | Climate perceptions index | EF carbon footprint | CO₂ emissions from fossil fuel combustion and cement production (tCO_2_/cap) | CO₂ emissions embodied in imports (tCO₂/cap) | Carbon pricing score at EUR60/  tCO₂ (%) |
|  | **Model 1** | **Model 2** | **Model 3** | **Model 4** | **Model 5** | **Model 6** | **Model 7** | **Model 8** | **Model 9** | **Model 10** | **Model 11** | **Model 12** |
| Mean | 82.567 | 54.842 | 67.333 | 63.300 | 58.897 | 63.167 | 42.207 | 55.355 | 2.036 | 6.819 | 2.931 | 4.398 |
| Median | 60.500 | 52.455 | 66.000 | 63.000 | 57.950 | 62.500 | 37.350 | 57.026 | 1.673 | 6.510 | 2.835 | 36.021 |
| Maximum | 186.000 | 71.336 | 81.000 | 78.000 | 90.000 | 153.000 | 80.900 | 68.286 | 5.773 | 15.368 | 8.606 | 69.313 |
| Minimum | 1.000 | 37.536 | 55.000 | 49.000 | 31.000 | 2.000 | 0.000 | 36.202 | 0.220 | 1.751 | 0.375 | 16.883 |
| Standard Deviation | 57.094 | 10.087 | 7.434 | 8.968 | 13.508 | 41.568 | 22.604 | 8.384 | 1.452 | 3.266 | 1.690 | 13.710 |
| Skewness | 0.297 | 0.154 | 0.355 | 0.136 | 0.067 | 0.310 | 0.050 | -0.674 | 0.971 | 1.128 | 1.131 | 0.404 |
| Kurtosis | 1.657 | 1.755 | 2.009 | 1.636 | 2.944 | 2.369 | 2.239 | 2.515 | 3.294 | 3.743 | 4.809 | 2.451 |
| Observations | 169 | 169 | 169 | 169 | 169 | 169 | 169 | 169 | 169 | 169 | 169 | 169 |

Table S6. Results of goodness-of-fit testing of C^3^S models.

| **Independent countries’ variables** | **Dependent countries’ climate change variables** | | | | | | | | | | | |
| --- | --- | --- | --- | --- | --- | --- | --- | --- | --- | --- | --- | --- |
|  | Energy use rank | ND-GAIN index | Public belief in the climate emergency | Country who say we should do everything necessary | EPI climate change | EPI climate change rank | EPI greenhouse gas emissions per capita | Climate perceptions index | EF carbon footprint | CO₂ emissions from fossil fuel combustion and cement production (tCO2/cap) | CO₂ emissions embodied in imports (tCO₂/cap) | Carbon pricing score at EUR60/  tCO₂ (%) |
|  | **Model 1** | **Model 2** | **Model 3** | **Model 4** | **Model 5** | **Model 6** | **Model 7** | **Model 8** | **Model 9** | **Model 10** | **Model 11** | **Model 12** |
| GDP per capita | -.088499  (.6419) | .89290***  (.0000) | .543024**  (.0019) | .69617***  (.0000) | .77017***  (.0000) | -.7925***  (.0000) | -.7058***  (.0000) | .4166*  (.0220) | .87218***  (.0000) | .451213  .0051 | .528678  .0008 | .556334  .0003 |
| GDP per capita in PPP | -.13244  (.4854) | .9291***  (.0000) | .51051**  (.0039) | .7428***  (.0000) | .7729***  (.0000) | -.7967***  (.0000) | -.7501***  (.0000) | .4989**  (.0050) | .8711***  (.0000) | .4759**  (.0029) | .5269***  (.0008) | .5601***  (.0003) |
| Ease of doing business ranking | .237422  (.2065) | -.8353***  (.0000) | -.4751**  (.0080) | -.4880**  (.0062) | -.6880***  (.0000) | .6919***  (.0000) | .4864**  (.0064) | -.3721*  (.0429) | -.6665***  (.0001) | -.2695  (.1066) | -.4161  (.0104) | .0436  (.7975) |
| Corruption perceptions index | -.121627  (.5220) | .9501***  (.0000) | .5970***  (.0005) | .6791***  (.0000) | .8188***  (.0000) | -.8281***  (.0000) | -.6323***  (.0002) | .4863**  (.0064) | .7738***  (.0000) | .3562  (.0304) | .5364***  (.0006) | .2998  (.0714) |
| Human development index | -.105240  (.5799) | .9235***  (.0000) | .4891**  (.0061) | .7193***  (.0000) | .7073***  (.0000) | -.7294***  (.0000) | -.7479***  (.0000) | .5835***  (.0007) | .7719***  (.0000) | .4756**  (.0029) | .5616***  (.0003) | .4297**  (.0079) |
| Global gender gap | -.138189  (.4665) | .5860***  (.0007) | .40003*  (.0285) | .6267***  (.0002) | .6173***  (.0003) | -.6295***  (.0002) | -.4549*  (.0115) | .6582***  (.0001) | .4966**  (.0052) | -.1090  (.5207) | .4875**  (.0022) | .3302  (.0459) |
| Happiness index | -.045199  (.8125) | .7988***  (.0000) | .4832**  (.0068) | .7335***  (.0000) | .7252***  (.0000) | -.7319***  (.0000) | -.6972***  (.0000) | .5604**  (.0013) | .7050***  (.0000) | .2913  (.0802) | .5228***  (.0009) | .3654  (.0262) |
| Environmental performance index | -.197300  (.2960) | .9384***  (.0000) | .6408***  (.0001) | .7551***  (.0000) | .8655***  (.0000) | -.8689***  (.0000) | -.6806***  (.0000) | .4769**  (.0077) | .7492***  (.0000) | .2692  (.1071) | .4200*  (.0096) | .5488***  (.0004) |
| Freedom and control | -.031427  (.8691) | -.6937***  (.0000) | -.5331**  (.0024) | -.7452***  (.0000) | -.6942***  (.0000) | .71921***(.0000) | .5419**  (.0020) | -.7369***  (.0000) | -.6063***  (.0004) | -.2276  (.1754) | -.4149  (.0107) | -.3357  (.0422) |
| Economic freedom | -.040050  (.8336) | .8114***  (.0000) | .4833**  (.0068) | .5748***  (.0009) | .6386***  (.0001) | -.6418***  (.0001) | -.5286***  (.0027) | .3897*  (.0333) | .7039***  (.0000) | .4362*  (.0070) | .4981*  (.0017) | .0069  (.9672) |
| Democracy index | .029983  (.8750) | .7788***  (.0000) | .5178**  (.0034) | .7096***  (.0000) | .6714***  (.0000) | -.6898***  (.0000) | -.5767***  (.0008) | .7291***  (.0000) | .6688***  (.0001) | .3532  (.0320) | .4527*  (.0049) | .3654  (.0261) |
| Unemployment rate | .196718  (.2975) | -.200938  (.2870) | .008659  (.9638) | .130683  (.4912) | -.052883  (.7814) | .019544  (.9184) | -.070274  (.7121) | -.085211  (.6544) | -.031414  (.8691) | -.2693  (.1069) | -.2907  (.0808) | -.1927  (.2531) |
| Healthy life expectancy | -.124962  (.5106) | .8414***  (.0000) | .43439*  (.0165) | .5600**  (.0013) | .6065***  (.0004) | -.6237***  (.0002) | -.5700**  (.0010) | .5211**  (.0031) | .5761***  (.0009) | .2466  (.1410) | .1245  (.4627) | .4637*  (.0038) |
| Fragile state index | .013700  (.9427) | -.9141***  (.0000) | -.5126**  (.0038) | -.7036***  (.0000) | -.7917***  (.0000) | .8235***  (.0000) | .7132***  (.0000) | -.5705**  (.0010) | -.780***  (.0000) | -.3882  (.0176) | -.5263***  (.0008) | -.4291*  (.0080) |
| Economic decline index | .007494  (.9686) | -.784***  (.0000) | -.298727  (.1088) | -.5015**  (.0047) | -.4450*  (.0137) | .4776**  (.0076) | .6191***  (.0003) | -.4263*  (.0188) | -.7072***  (.0000) | -.5724***  (.0002) | -.4536**  (.0048) | -.2300  (.1707) |
| R^2^ | .449 | .954 | .514 | .786 | .773 | .779 | .740 | .569 | .753 | .541 | .762 | .641 |
| Adjusted R^2^ | .394 | .950 | .166 | .634 | .750 | .756 | .713 | .494 | .725 | .494 | .733 | .384 |
| F | 8.190*** | 204.597*** | 1.479 | 5.151*** | 32.716*** | 33.900*** | 27.327*** | 7.574*** | 26.680*** | 11.694 | 27.044 | 2.498 |

Standardized beta coefficients: *significant at - p<0.1, **significant at p<0.01 and ***significant at α = p<0.001.

Table S7. Standardized coefficient beta values for the dependent climate change variables.

| **Dependent**  **countries’**  **climate**  **change**  **variables**    **Independent**  **countries’**  **variables** | | **Standardized coefficients' beta values of the dependent variables** | | | | | | | | | | | |
| --- | --- | --- | --- | --- | --- | --- | --- | --- | --- | --- | --- | --- | --- |
|  |  | Energy use rank | ND-GAIN index | Public belief in the climate emergency | Country who say we should do everything necessary | EPI climate change | EPI climate change rank | EPI greenhouse gas emissions per capita | Climate perceptions Index | EF carbon footprint | CO₂ emissions from fossil fuel combustion and cement production (tCO2/cap) | CO₂ emissions embodied in imports (tCO₂/cap) | Carbon pricing score at EUR60/  tCO₂ (%) |
|  |  | **Model 1** | **Model 2** | **Model 3** | **Model 4** | **Model 5** | **Model 6** | **Model 7** | **Model 8** | **Model 9** | **Model 10** | **Model 11** | **Model 12** |
| 1 | GDP per capita | .4327 | .1587* | .0420 | -.3221 | .1191 | -.0342 | .5234** | -.3477 | -.2868 | -.4764 | -.1515 | .1909 |
| 2 | GDP per capita in PPP | -.5064 | -.1288 | -.0349 | 1.2075 | -.2060 | .0784 | -.7567*** | .2025 | 1.0549*** | 1.0153** | .8264** | .3454 |
| 3 | Ease of doing business ranking | .1946 | -.2318*** | .1127 | .3083 | -.2050* | .1823 | .0583 | -.2829 | .0297 | .0062 | .1052 | .2652 |
| 4 | Corruption perceptions index | -.0739 | .2187*** | -.0156 | .3774 | -.2618* | .2945** | -.0482 | .1579 | -.0346 | -.0735 | .1826 | -.4428 |
| 5 | Human development index | -.2361 | .2760*** | -.3855 | -.5427 | .0137 | -.0565 | -.3571** | -.1856 | .0367 | .1641 | -.0120 | .2360 |
| 6 | Global gender gap | -.0010 | -.0232 | .0971 | .2608 | .0021 | -.0104 | -.0233 | .3876** | -.0891 | -.0849 | .1234* | .6259* |
| 7 | Happiness index | .2407* | .0386 | -.3517 | .2709 | -.0161 | .0402 | -.1223 | -.0705 | .0423 | .1218 | .0237 | -.6090 |
| 8 | Environmental performance index | -.6811*** | .0731 | 1.1259 | -.2345 | .9576*** | -.9330*** | -.1171 | -.2311 | -.1716 | -.3774** | -.0443 | .5398 |
| 9 | Freedom and control | -.1200 | .0742 | -.1214 | -.7541 | .0583 | -.0389 | -.1946 | -.8843** | .0058 | .0985 | -.0824 | .1985 |
| 10 | Economic freedom | .0459 | .0491 | .5016 | .3328 | -.0493 | .0484 | .2823** | -.3356* | .0407 | -.0182 | .2004* | -.0181 |
| 11 | Democracy index | .1037 | -.0595 | -.0478 | -.4605 | .1044 | -.0934 | .0308 | -.2131 | -.1729 | -.1836 | -.2327 | -.1268 |
| 12 | Unemployment rate | .2058** | .0616** | .1259 | .2793* | .1370** | -.1355** | -.1439** | -.0513 | .0787 | .0675 | -.0199 | -.1950 |
| 13 | Healthy life expectancy | .3524*** | .0632* | .0055 | .5312 | .0539 | -.0528 | .0766 | .3385* | .0085 | -.0533 | -.0472 | .2488 |
| 14 | Fragile state index | -.0350 | -.2573*** | .3081 | .7823 | -.1220 | .1455 | .2159 | .5764 | -.2479 | -.3580 | -.0740 | -.0878 |
| 15 | Economic decline index | -.0673 | .0126 | -.0763 | .0127 | .1966 | -.2149 | .1498 | -.4422 | -.1034 | -.1781 | -.0048 | -.0128 |

Standardized beta coefficients: *significant at - p<0.1, **significant at p<0.01 and ***significant at α = p<0.001.

Table S8. Weights of the impact in C^3^QL regression models calculated for the dependent climate change variables.

|  | **Dependent cities’**  **climate change**  **variables**  **Independent**  **cities’ variables** | **Global Gridded Model of Carbon Footprints (GGMCF) Footprint/cap(t CO2)** | **BASIC GHG emissions data for C40 cities, total emission per capita (tCO2e)** | Σ |
| --- | --- | --- | --- | --- |
|  |  | 1 | 2 | 3 |
| 1 | Quality of life index | 3.833 | 2.839 | 6.672 |
| 2 | Purchasing power index | 2.971 | 4.000 | 6.971 |
| 3 | Safety index | 0.288 | 0.699 | 0.987 |
| 4 | Health care index | 0.556 | 0.590 | 1.145 |
| 5 | Cost of living index | 3.970 | 0.757 | 4.727 |
| 6 | Property price to income ratio | 1.596 | 1.100 | 2.696 |
| 7 | Traffic commute time index | 0.727 | 0.604 | 1.332 |
| 8 | Pollution index | 1.864 | 1.389 | 3.253 |
| 9 | Climate index | 0.819 | 0.458 | 1.278 |

Table S9. Theoretical multiple criteria and regression analysis of the climate change policy alternatives in the 169 countries.

| **Effect size**  Statistical methods are applied to determine the statistical relationships between countries success and the climate change indicators | The sign ‘+/-’ indicates that either higher (+) or lower (-) value of a criterion improves/worsens the impact of a factor on the improvement of the climate change indicators | **Weights** | **Measuring units** | **Independent C^3^S model variables**  **a statistical evaluation of the countries success data according to the climate change indicators** | | | | | |
| --- | --- | --- | --- | --- | --- | --- | --- | --- | --- |
|  |  |  |  | **GDP per capita (a_1_), Q_1_** | **GDP per capita in PPP (a_2_), Q_2_** | **…** | **Environmental performance index (a_8_), Q_8_** | **…** | **Economic decline index (a_15_), Q_15_** |
| **1st C^3^S model (energy use rank model)** | | | | | | | | | |
| 1. Pearson’s correlation coefficient (\| r \|) | + | q^1^_1_ | Numbers | x^1^_11_ | x^1^_12_ | … | x^1^_18_ | … | x^1^_1 15_ |
| 1. Coefficient of determination (R^2^): The 15 independent variables explain the dependent variable under analysis in % | + | q^1^_2_ | Numbers | x^1^_21_ | x^1^_22_ | … | x^1^_28_ | … | x^1^_2 15_ |
| 1. Standardized beta coefficient (β) | + | q^1^_3_ | Estimates | x^1^_31_ | x^1^_32_ | … | x^1^_38_ | … | x^1^_3 15_ |
| 1. Standard deviation | - | q^1^_4_ | Values | x^1^_41_ | x^1^_42_ | … | x^1^_48_ | … | x^1^_4 15_ |
| 1. p values (probability level) | - | q^1^_5_ | Values | x^1^_51_ | x^1^_52_ | … | x^1^_58_ | … | x^1^_5 15_ |
| 6. The percentage increase of a climate change  indicator value caused by a 1% increase in  the country’s success indicator (P_id_) | + | q^1^_6_ | Values | x^1^_61_ | x^1^_62_ | … | x^1^_68_ | … | x^1^_6 15_ |
| **The values are pre-set by users and experts** | | | | | | | | | |
| 7. Country context | + | q^1^_7_ | The weights are pre-set by users and experts | x^1^_71_ | x^1^_72_ | … | x^1^_78_ | … | x^1^_7 15_ |
| 8. Practical significance | + | q^1^_8_ |  | x^1^_81_ | x^1^_82_ | … | x^1^_88_ | … | x^1^_8 15_ |
| 9. Indicators with low values | + | q^1^_9_ |  | x^1^_91_ | x^1^_92_ | … | x^1^_98_ | … | x^1^_9 15_ |
| **…** | | | | | | | | | |
| **12^th^ C^3^S Model (carbon pricing score at eur60/tCO₂ model)** | | | | | | | | | |
| 1. Pearson’s correlation coefficient (\| r \|) | + | q^12^_1_ | Numbers | x^12^_11_ | x^12^_12_ | … | x^12^_18_ | … | x^12^_1 15_ |
| 1. Coefficient of determination (R^2^): The 15 independent variables explain the dependent variable under analysis in % | + | q^12^_2_ | Numbers | x^12^_21_ | x^12^_22_ | … | x^12^_28_ | … | x^12^_2 15_ |
| 1. Standardized beta coefficient (β) | + | q^12^_3_ | Estimates | x^12^_31_ | x^12^_32_ | … | x^12^_38_ | … | x^12^_3 15_ |
| 1. Standard deviation | - | q^12^_4_ | Values | x^12^_41_ | x^12^_42_ | … | x^12^_48_ | … | x^12^_4 15_ |
| 1. p values (probability level) | - | q^12^_5_ | Values | x^12^_51_ | x^12^_52_ | … | x^12^_58_ | … | x^12^_5 15_ |
| 6. The percentage increase of a climate change  indicator value caused by a 1% increase in  the country’s success indicator (P_id_) | + | q^12^_6_ | Values | x^12^_61_ | x^12^_62_ | … | x^12^_68_ | … | x^12^_6 15_ |
| **The values are pre-set by users and experts** | | | | | | | | | |
| 7. Country context | + | q^12^_7_ | The weights are pre-set by users and experts | x^12^_71_ | x^12^_72_ | … | x^12^_78_ | … | x^12^_7 15_ |
| 8. Practical significance | + | q^12^_8_ |  | x^2^_81_ | x^12^_82_ | … | x^12^_88_ | … | x^12^_8 15_ |
| 9. Indicators with low values | + | q^12^_9_ |  | x^12^_91_ | x^12^_92_ | … | x^12^_98_ | … | x^12^_9 15_ |
| Significance | | | | P_1_ | P_2_ | … | P_8_ | … | P_15_ |
| Priority | | | | P_1_ | P_2_ | … | P_8_ | … | P_15_ |
| Utility degrees | | | | N_1_ | N_2_ | … | N_8_ | … | N_15_ |
